# Single-cell transcriptomes underscore genetically distinct tumor characteristics and microenvironment for hereditary kidney cancers

**DOI:** 10.1016/j.isci.2022.104463

**Published:** 2022-05-25

**Authors:** Ryosuke Jikuya, Koichi Murakami, Akira Nishiyama, Ikuma Kato, Mitsuko Furuya, Jun Nakabayashi, Jordan A. Ramilowski, Haruka Hamanoue, Kazuhiro Maejima, Masashi Fujita, Taku Mitome, Shinji Ohtake, Go Noguchi, Sachi Kawaura, Hisakazu Odaka, Takashi Kawahara, Mitsuru Komeya, Risa Shinoki, Daiki Ueno, Hiroki Ito, Yusuke Ito, Kentaro Muraoka, Narihiko Hayashi, Keiichi Kondo, Noboru Nakaigawa, Koji Hatano, Masaya Baba, Toshio Suda, Tatsuhiko Kodama, Satoshi Fujii, Kazuhide Makiyama, Masahiro Yao, Brian M. Shuch, Laura S. Schmidt, W. Marston Linehan, Hidewaki Nakagawa, Tomohiko Tamura, Hisashi Hasumi

**Affiliations:** 1 Department of Urology, Yokohama City University Graduate School of Medicine, Yokohama, Kanagawa 236-0004, Japan; 2 Laboratory for Cancer Genomics, RIKEN Center for Integrative Medical Sciences, Yokohama, Kanagawa 230-0045, Japan; 3 Department of Immunology, Yokohama City University Graduate School of Medicine, Yokohama, Kanagawa 236-0004, Japan; 4 Advanced Medical Research Center, Yokohama City University, Yokohama, Kanagawa 236-0004, Japan; 5 Department of Molecular Pathology, Yokohama City University Graduate School of Medicine, Yokohama, Kanagawa 236-0004, Japan; 6 Clinical Genetics Department, Yokohama City University Graduate School of Medicine, Yokohama, Kanagawa 236-0004, Japan; 7 Department of Urology, Osaka University Graduate School of Medicine, Osaka 565-0871 Japan; 8 Laboratory of Cancer Metabolism, International Research Center for Medical Sciences, Kumamoto University, Kumamoto 860-0811, Japan; 9 Laboratory for Systems Biology and Medicine, Research Center for Advanced Science and Technology, University of Tokyo, Tokyo 153-8904, Japan; 10 Institute of Urologic Oncology, UCLA School of Medicine, Los Angeles, CA 90095, USA; 11 Urologic Oncology Branch, Center for Cancer Research, National Cancer Institute, National Institutes of Health, Bethesda, MD 20892, USA; 12 Basic Science Program, Frederick National Laboratory for Cancer Research, Frederick, MD 21702, USA

**Keywords:** Oncology, Microenvironment, Human specimen, Cancer systems biology, Cancer

## Abstract

Our understanding of how each hereditary kidney cancer adapts to its tissue microenvironment is incomplete. Here, we present single-cell transcriptomes of 108,342 cells from patient specimens including from six hereditary kidney cancers. The transcriptomes displayed distinct characteristics of the cell of origin and unique tissue microenvironment for each hereditary kidney cancer. Of note, hereditary leiomyomatosis and renal cell carcinoma (HLRCC)-associated kidney cancer retained some characteristics of proximal tubules, which were completely lost in lymph node metastases and present as an avascular tumor with suppressed T cells and TREM2-high macrophages, leading to immune tolerance. Birt-Hogg-Dubé (BHD)-associated kidney cancer exhibited transcriptomic intratumor heterogeneity (tITH) with increased characteristics of intercalated cells of the collecting duct and upregulation of FOXI1-driven genes, a critical transcription factor for collecting duct differentiation. These findings facilitate our understanding of how hereditary kidney cancers adapt to their tissue microenvironment.

## Highlights

• scRNA-seq displayed genetically defined characteristics of hereditary kidney cancer
• scRNA-seq revealed unique tissue microenvironment of each hereditary kidney cancer
• BHD-associated kidney cancer showed transcriptomic intratumor heterogeneity (tITH)
• BHD-associated kidney cancer showed intercalated cell characteristics driven by FOXI1

## Graphical abstract

**Figure:**
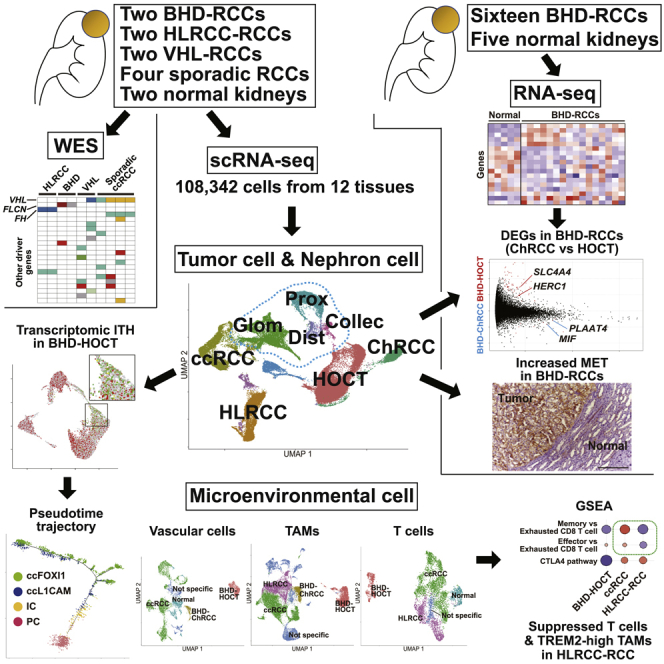

## Introduction

Dysregulation of metabolism and the epigenome drives renal tumorigenesis, because most kidney cancer-associated genes are either metabolic or chromatin remodeling genes (Hasumi et al., 2018; Hasumi and Yao, 2018; Linehan et al., 2019). Kidney cancers arise from various types of cells that constitute the nephron, which develop from mesenchymal cells arising from the intermediate mesoderm interacting with epithelial cells from the invading ureteric bud (Maciaszek et al., 2020). The diversity of kidney cancer-associated genes and the variety of originating cell types underscore the complexity of renal tumorigenesis.

Hereditary kidney cancer accounts for 5–8% of all kidney cancers and loss of each causative gene develops distinct histological subtypes of kidney cancer as well as systemic manifestations in particular organs (Ball and Shuch, 2019). To date, thirteen hereditary kidney cancer syndromes have been described and mechanistic insights into these rare disorders driven by loss of kidney cancer-associated genes have provided a foundation for the development of novel therapeutics and diagnostics for hereditary kidney cancer and for sporadic kidney cancer as well (Hasumi and Yao, 2018; Linehan et al., 2019).

Each hereditary kidney cancer is a uniquely different disease in which aggressiveness, including tumor proliferation and invasion, is determined by the causative gene. The average growth rate of HLRCC-associated kidney cancer is estimated to be 1.06 cm per year, whereas the median growth rate of von Hippel-Lindau (VHL)-associated kidney cancer is 0.37 cm per year and that of BHD-associated kidney cancer is 0.1 cm per year (Ball et al., 2020; Paschall et al., 2020). HLRCC-associated kidney cancer has a propensity to metastasize when the primary tumor is small (as small as 0.5 cm), underscoring the aggressive and invasive nature of the tumor (Grubb et al., 2007; Linehan et al., 2019).

Although much has been learned about tumorigenesis associated with deficiency of each of these causative genes, our understanding of how each hereditary kidney cancer adapts to its tissue microenvironment is incomplete. Recent advances in single-cell transcriptome analysis have facilitated further understanding of the precise molecular characteristics of tumor cells at the single-cell level and how tumor cells adapt to their tissue microenvironment. In this study, we delineate tumor characteristics and the tissue microenvironment of genetically defined hereditary kidney cancers using single-cell RNA-sequencing.

## Results

### Single-cell transcriptomes exhibit an association of each hereditary kidney cancer with its cell of origin

**Figure 1:**
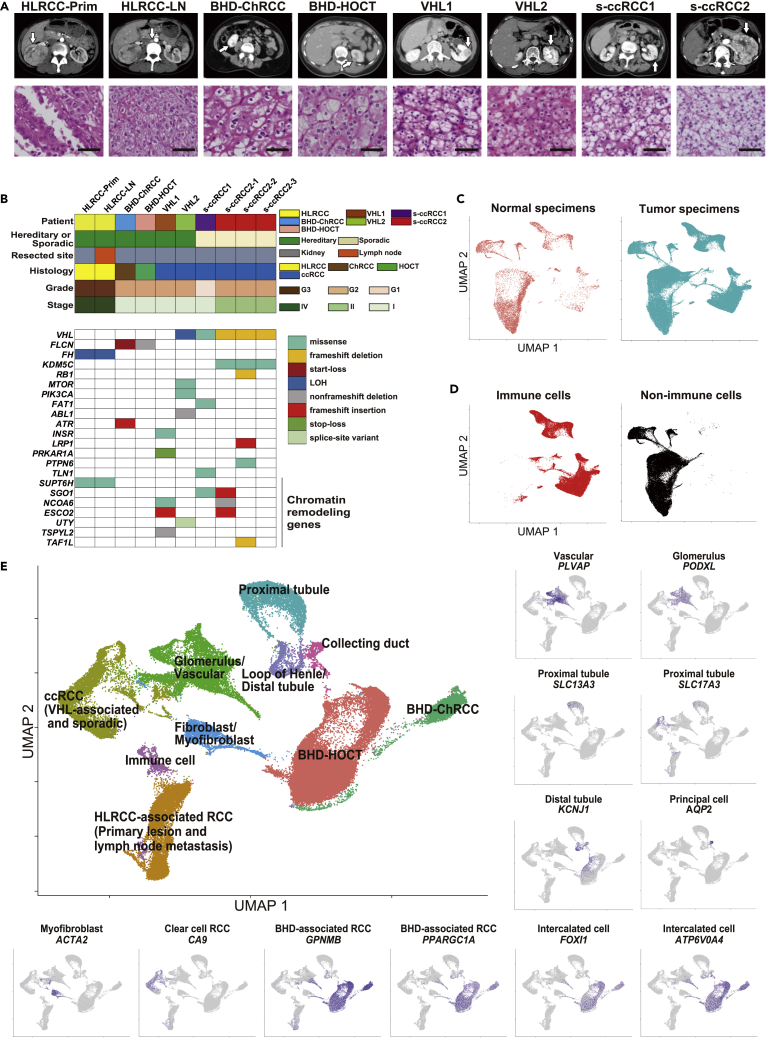
Single-cell transcriptome exhibits an association of each hereditary kidney cancer with its cell of origin (A) Computed tomography and hematoxylin-eosin staining of kidney cancers analyzed in this study. Arrows indicate tumors. Image shows a 400× magnification of hematoxylin and eosin staining. Scale bars represent 50 μm. (B) Clinical information and somatic variants identified by whole exome sequencing. (C) Uniform manifold approximation and projection (UMAP) plots of cells from two normal kidneys (left) and those from ten kidney cancers (right). (D) UMAP plots of CD45 positive immune cells (left) and CD45 negative nonimmune cells (right) from twelve specimens. (E) UMAP plot of CD45 negative nonimmune cells from all twelve specimens annotated with gene markers, whose expressions are shown in surrounding figures and Figure S1F. Some residual immune cells which are seen as dark purple dots in CD45 negative cluster 0, 2, 3, 4, 6, 10, 12, 13, 15, 17, 22, 23, and 25 in the upper panel of Figure S1C were re-clustered as immune cells. Abbreviations: BHD-ChRCC, BHD-associated chromophobe renal cell carcinoma; BHD-HOCT, BHD-associated hybrid oncocytic chromophobe tumor; ccRCC, clear cell renal cell carcinoma; HLRCC-LN, lymph node metastasis of HLRCC-associated kidney cancer; HLRCC-Prim, primary lesion of HLRCC-associated kidney cancer; s-ccRCC, sporadic clear cell renal cell carcinoma.

To explore how the tumor cell of hereditary kidney cancer adapts to its tissue microenvironment, we conducted single-cell RNA sequencing of twelve surgically resected specimens from seven patients including one BHD-associated hybrid oncocytic chromophobe tumor (HOCT), one BHD-associated chromophobe renal cell carcinoma, one primary lesion and one lymph node metastasis from HLRCC-associated kidney cancer, two VHL-associated kidney cancers, one sporadic clear cell renal cell carcinoma, 3 intratumoral samples from a second sporadic clear cell renal cell carcinomas, and two normal kidney tissues (Figure 1A). To define genetic alterations of those cancers, we performed whole exome sequencing, which revealed second hit alterations of the hereditary kidney cancer-causative genes as well as somatic mutations in the sporadic clear cell renal cell carcinomas (Figure 1B).

We obtained the single-cell transcriptomes of 108,342 cells from these twelve tissues and first divided into 46,890 immune cells and 61,452 nonimmune cells using CD45, an immune cell marker (Figures 1C and 1D and S1A–S1C). Nonimmune cells were annotated into cell clusters using previously reported marker genes for intercalated or principal cells of the collecting duct, distal tubules, loop of Henle, proximal tubules, glomerulus/vascular, and kidney cancers (Figures 1E and S1D–S1F) (Aird, 2007; Cancer Genome Atlas Research, 2013; Chabardes-Garonne et al., 2003; Habuka et al., 2014; Han and Amar, 2004; Lake et al., 2019; LeBleu et al., 2013; Lee et al., 2015; Nawroth et al., 2002; Schmidt, 2013; Wang et al., 2017). Interestingly, normal kidney cells representing intercalated or principal cells of the collecting duct, distal tubules, loop of Henle, proximal tubules, and glomerulus/vascular were clustered side-by-side representing tubules and capillaries of the nephron. Cells from VHL-associated kidney cancers and sporadic clear cell renal cell carcinomas were clustered close to glomerulus/vascular, suggesting that those cancers and glomerulus/vascular might share a portion of their transcriptomic profile, and therefore, those cancers may arise from glomerulus/vascular cells(Chen et al., 2016; Gu et al., 2017). Cells from the primary lesion and lymph node metastasis of HLRCC-associated kidney cancer formed an independent cluster distant from nephron cells; however, weak expressions of *solute carrier family 17 member 3* (*SLC17A3*), a marker gene for proximal tubule, suggest that HLRCC-associated kidney cancer may have originated from the proximal tubule. Importantly, cells of BHD-associated kidney cancer were clustered in the vicinity of collecting duct, suggesting that BHD-associated kidney cancer might have originated from collecting duct (Figures 1E and S1D–S1F and Dataset S1).

### Single-cell transcriptome delineates characteristic tissue microenvironment of each hereditary kidney cancer

**Figure 2:**
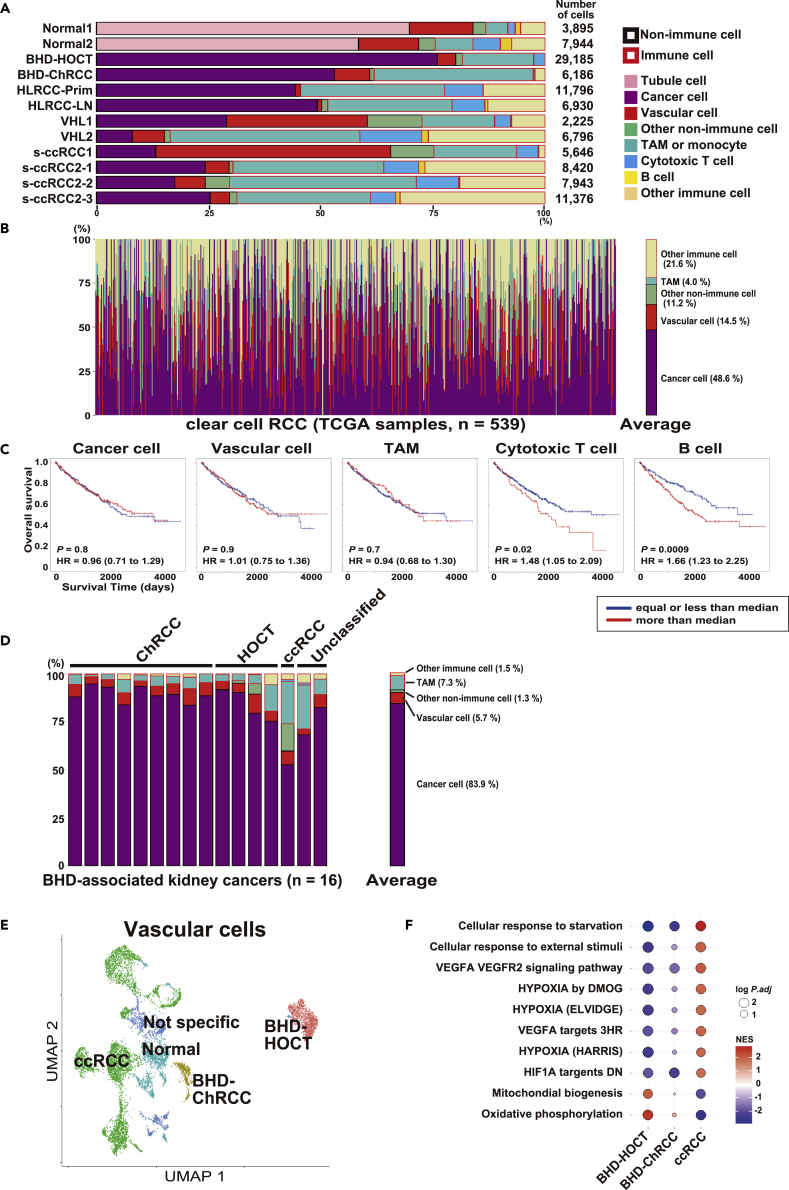
Single-cell transcriptome delineates characteristic tissue microenvironment of each hereditary kidney cancer (A) Bar plot of cell type composition. Horizontal axis means ratio of cell number of each cell type per total cell number in each specimen. TAM, tumor-associated macrophage. (B) Predicted cell type composition of 539 ccRCC samples from The Cancer Genome Atlas (TCGA) project using deconvolution pipelines. (C) Overall survival of 539 ccRCC patients from TCGA project based on predicted cell type composition. Patients were divided into two groups by the median ratio of cell number of each cell type per total cell number. *p* values from 2-sided log rank test, and the hazard ratio with 95% confidence interval is shown. (D) Predicted cell type composition of 16 BHD-associated kidney cancers using deconvolution pipelines. (E) UMAP plot of vascular cells from all of the twelve specimens. The cell number in each cluster is as follows: 462 vascular cells for BHD-ChRCC, 1,181 vascular cells for BHD-HOCT, 4,920 vascular cells for ccRCC, 1,462 vascular cells for normal kidney (Normal), and 1,081 vascular cells for nonspecific cluster (Not specific) cells. (F) Gene Set Enrichment Analysis (GSEA) comparing vascular cells of BHD-HOCT, BHD-ChRCC, and ccRCC (VHL-associated and sporadic). Abbreviations: BHD-ChRCC, BHD-associated chromophobe renal cell carcinoma; BHD-HOCT, BHD-associated hybrid oncocytic chromophobe tumor; ccRCC, clear cell renal cell carcinoma; ChRCC, chromophobe renal cell carcinoma; HLRCC-LN, lymph node metastasis of HLRCC-associated kidney cancer; HLRCC-Prim, primary lesion of HLRCC-associated kidney cancer; HOCT, hybrid oncocytic chromophobe tumor; HR, hazard ratio; s-ccRCC, sporadic clear cell renal cell carcinoma; TAM, tumor associated macrophage; Unclassified, unclassified renal cell carcinoma.

A total of 46,890 immune cells were annotated into each immune cell cluster based on previously established gene markers (Figures S1G–S1J) (Zhang et al., 2019). Cell type composition of each tumor revealed that the large HLRCC-associated kidney cancer (7 cm) had poor vascularity, implying that in this case, HLRCC-associated kidney cancer and its tissue microenvironment may be continuously exposed to hypoxia and low nutrition (Figure 2A). Analyzing bulk RNA-seq data with a deconvolution pipeline, we predicted cell type compositions of 539 clear cell renal carcinomas investigated in the TCGA project as well as those of 16 BHD-associated kidney cancers analyzed in this study (Figures S2A–S2D and Dataset S2) (Cancer Genome Atlas Research, 2013; Newman et al., 2019). Predicted cell type compositions of clear cell renal carcinomas in the The Cancer Genome Atlas (TCGA) project were diverse and ratios of cytotoxic T cell and B cell numbers per total cell number were found to be prognostic factors for overall survival (Figures 2B and 2C). On the other hand, predicted cell type compositions of BHD-associated kidney cancers were similar across all tumors, consistent with our previous notion that BHD-associated kidney cancer is genetically uniform with very few driver variants other than variants in the *folliculin* (*FLCN*) gene (Figure 2D) (Hasumi et al., 2018). The average ratio of cancer cell number per total cell number in clear cell renal carcinomas from the TCGA project was 48.6%, compared with 83.9% for BHD-associated kidney cancer, suggesting that BHD-associated kidney cancers may be indolent with a smaller number of non-tumor infiltrating cells and vascular cells. The molecular profiling of vascular cells in clear cell renal carcinomas analyzed by single-cell sequencing exhibited increased hypoxia signaling, supporting that the VHL-Hypoxia-inducible factor (HIF) pathway is dysregulated in these tumors (Figures 2E and 2F and S2E). Interestingly, vascular cells in BHD-associated HOCT demonstrated increased oxidative phosphorylation without an upregulated HIF-Vascular endothelial growth factor (VEGF) signature compared to clear cell renal cell carcinomas, suggesting that receptor tyrosine kinase inhibitors targeting tumor angiogenesis may not be effective in BHD-associated kidney cancer and a totally different therapeutical approach may be required for targeting vascular cells in BHD-associated kidney cancer.

### HLRCC-associated kidney cancer harbors suppressed T cells and *TREM2*-high tumor associated macrophages (TAMs)

**Figure 3:**
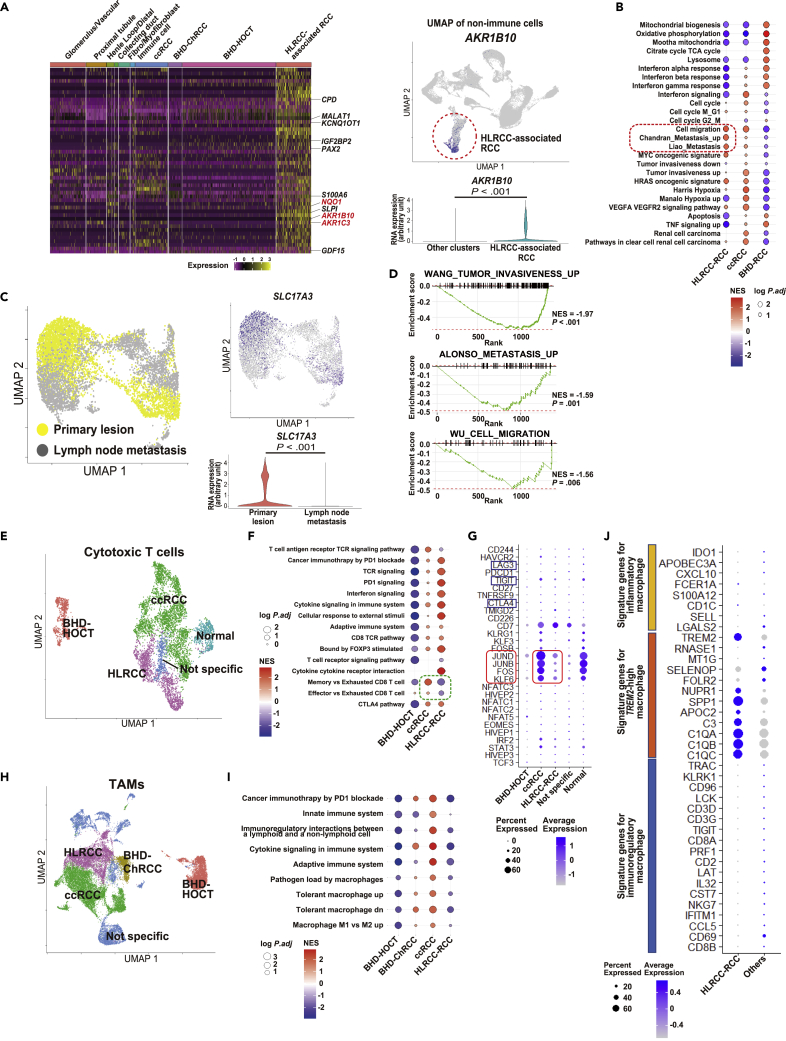
HLRCC-associated kidney cancer harbors suppressed T cells and *TREM2*-high tumor associated macrophages (TAMs) (A) Heatmap of top 50 highly expressed genes in HLRCC-associated kidney cancer. Red highlighted genes are associated with the KEAP1-NRF2 axis (left). Expression of *AKR1B10*, one of the known genes associated with KEAP1-NRF2 axis, on the UMAP plot and violin plot of non-immune cells in all the tumors (right). *p* values are from 2-sided Welch’s two sample *t*-tests. (B) GSEA results comparing tumor cells of HLRCC-associated kidney cancer (HLRCC-RCC), ccRCC (VHL-associated and sporadic), and BHD-associated kidney cancers (BHD-RCC). (C) UMAP plot of tumor cells from primary lesion and lymph node metastasis of HLRCC-associated kidney cancer (left). Expressions of *SLC17A3*, a proximal tubule marker in the left UMAP plot (right). (D) GSEA results comparing tumor cells of primary lesion and those of lymph node metastasis of HLRCC-associated kidney cancer. (E) UMAP plot of cytotoxic T cells from all twelve specimens colored by each group. The cell number in each cluster is as follows: 725 cytotoxic T cells for BHD-HOCT, 3,101 cytotoxic T cells for ccRCC (VHL-associated and sporadic), 1,357 cytotoxic T cells for HLRCC, 576 cytotoxic T cells for normal kidney (Normal), and 365 cytotoxic T cells for non-specific cluster (Not specific). (F) GSEA results comparing cytotoxic T cells of BHD-HOCT, ccRCC, and HLRCC-RCC. (G) Dot plot of marker genes for T cell homeostasis and differentiation (red squared genes) and T cell exhaustion markers (blue squared genes). (H) UMAP plot of TAMs from all twelve specimens colored by each group. The cell number in each cluster is as follows: 1,737 TAMs for BHD-ChRCC, 4,625 TAMs for BHD-HOCT, 9,213 TAMs for ccRCC, 5,395 TAMs for HLRCC, and 6,234 TAMs for nonspecific cell cluster (Not specific). (I) GSEA results comparing TAMs of BHD-HOCT, BHD-associated chromophobe renal cell carcinoma (BHD-ChRCC), ccRCC (VHL-associated and sporadic), and HLRCC-RCC. (J) Dot plot of signature gene expressions for various types of macrophages. Others include BHD-associated kidney cancers and ccRCC (VHL-associated and sporadic). Abbreviations: BHD-ChRCC, BHD-associated chromophobe renal cell carcinoma; BHD-HOCT, BHD-associated hybrid oncocytic chromophobe tumor; BHD-RCC, BHD-associated renal cell carcinoma; ccRCC, clear cell renal cell carcinoma; HLRCC-RCC, HLRCC-associated renal cell carcinoma; s-ccRCC, sporadic clear cell renal cell carcinoma; TAM, tumor associated macrophage.

To elucidate the molecular characteristics of HLRCC-associated kidney cancer, we mined the single-cell sequencing data to determine the gene expression profile. We found that *aldo-keto reductase family 1 member B10* (*AKR1B10*), *NAD(P)H quinone dehydrogenase 1* (*NQO1*), and *aldo-keto reductase family 1 member C3* (*AKR1C3*) are highly expressed in HLRCC-associated kidney cancer relative to the other cancer types, all of which have been reported to be highly expressed in HLRCC-associated kidney cancer in association with a dysregulated Kelch-like ECH-associated protein 1 (KEAP1)-nuclear factor erythroid 2-related factor 2 (NRF2) axis (Figure 3A) (Ooi et al., 2011). In addition, we found that *carboxypeptidase D* (*CPD*), *growth Differentiation Factor 15* (*GDF15*), *S100 calcium binding protein A6* (*S100A6*), secretory leukocyte peptidase inhibitor (*SLP1*), *paired box 2* (*PAX2*), *KCNQ1 opposite strand/antisense transcript 1* (*KCNQ1OT1*), *metastasis associated in lung adenocarcinoma transcript-1* (*MALAT1*), and *insulin like growth factor 2 mRNA binding protein 2* (*IGF2BP2*) are highly expressed in HLRCC-associated kidney cancer relative to the other cancer types, all of which have been reported to be expressed in highly aggressive cancers with poor prognosis (Chen et al., 2015; Feng et al., 2020; Gao et al., 2021; Han et al., 2020; Miyazaki et al., 2019; Munn and Garkavtsev, 2018; Shi et al., 2019; Wang et al., 2020; Ye et al., 2021; Yi et al., 2021; Zhang et al., 2021a). Using Gene Set Enrichment Analysis (GSEA), we compared three types of hereditary kidney cancers and found that gene sets related to cell migration and metastasis were enriched in HLRCC-associated kidney cancer, consistent with the fact that this highly aggressive kidney cancer subtype has a propensity to metastasize from a small primary lesion (Figure 3B).

Because the primary lesion and lymph node metastasis of HLRCC-associated kidney cancer were clustered in different clusters, we investigated their differentially expressed genes and found that the expression of *SLC17A3*—a proximal tubule marker—was lost in the lymph node metastasis (Figure 3C) (Jutabha et al., 2011). GSEA analysis revealed that gene sets related to tumor invasiveness, metastasis, and cell migration were enriched in the lymph node metastasis compared to the primary lesion, suggesting that the HLRCC primary tumor may need to modulate its gene expression profile to adapt to the lymph node tissue microenvironment (Figure 3D).

Next, we examined molecular profiling of cytotoxic T cells and tumor-associated macrophages (TAMs). Cytotoxic T cells in HLRCC-associated kidney cancer appeared to be suppressed with suppressed TAMs as well (Figures 3E–3I and S2F and S2G). In addition, expressions of signature genes for *triggering receptor expressed on myeloid cells 2* (*TREM2*)-high TAMs which are associated with poor prognosis, including *secreted phosphoprotein 1* (*SPP1*) that acts as an immune checkpoint to suppress T cell function, were enriched in HLRCC-associated kidney cancer, further suggesting that suppressed TAMs in HLRCC-associated kidney cancer may give rise to immune tolerance (Figure 3J) (Hu et al., 2020; Klement et al., 2018; Molgora et al., 2020; Xiong et al., 2020).

### BHD-associated kidney cancer exhibits transcriptomic intratumor heterogeneity and increased intercalated cell characteristics with upregulation of FOXI1-driven genes

**Figure 4:**
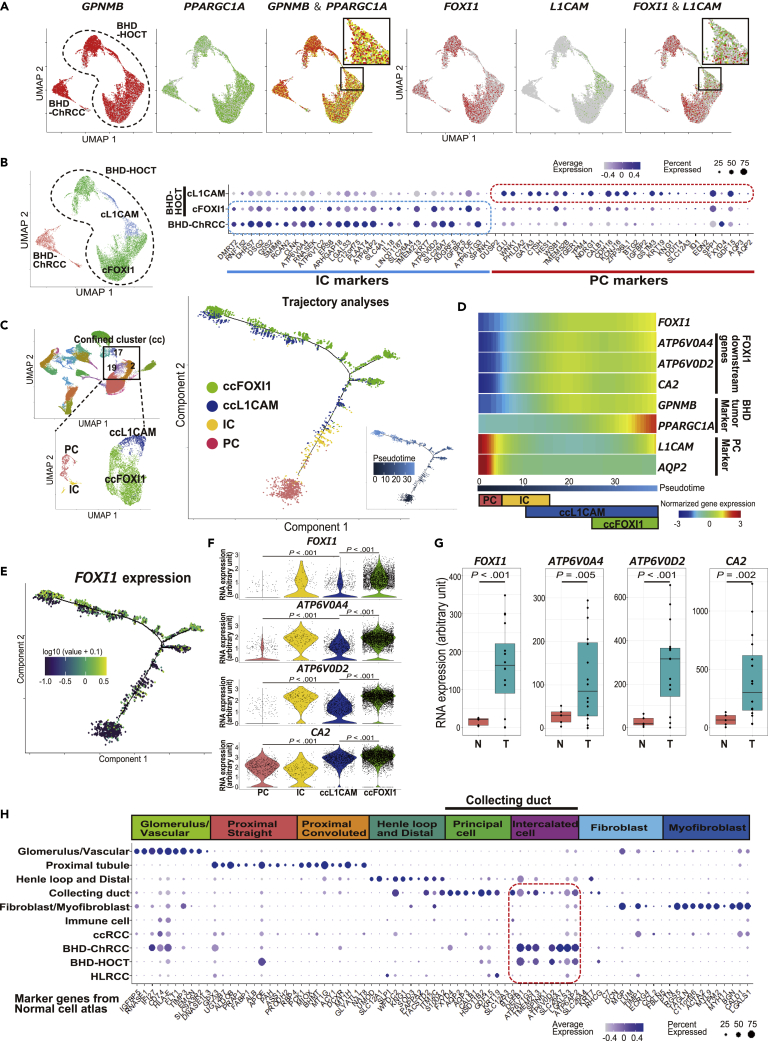
BHD-associated kidney cancer exhibits a transcriptomic intratumor heterogeneity (tITH) and increased intercalated cell characteristics with upregulated FOXI1-driven genes (A) UMAP plots of tumor cells of BHD-associated HOCT (BHD-HOCT) and chromophobe renal cell carcinoma (BHD-ChRCC) colored by expressions of each gene. Yellow colored cells express both genes. (B) UMAP plot of tumor in each cell of BHD-ChRCC and BHD-HOCT divided into two clusters; a cluster of *L1CAM* expressing cells (cL1CAM) and that of *FOXI1* expressing cells (cFOXI1) (left). Bubble chart of expressions of marker genes for intercalated cell (IC) and principal cell (PC) in each cluster; normal cell atlas was created using transcriptomes of two normal kidneys in this study and expressions of top thirteen marker genes from our normal cell atlas are indicated (right) (Figure S3 and Dataset S3). (C) Trajectory analysis of BHD-associated HOCT and benign collecting duct (right). For this trajectory analysis, clusters in Figure 4B were further narrowed down (confined) in accordance with an upper limit of our PC processing speed; a confined cluster of *L1CAM* expressing cells (ccL1CAM) and a confined cluster of *FOXI1* expressing cells (ccFOXI1) (left). (D) Gradient expressions of *FOXI1* and its downstream genes in the trajectory of Figure 4C. (E) *FOXI1* expressions in the trajectory of Figure 4C. (F) Violin plots of *FOXI1* and its downstream gene expressions in each cluster. *p* values from 2-sided Welch’s two sample *t*-test. (G) Boxplot of *FOXI1* and its downstream gene expressions. N, normal kidney (n = 5). T, BHD-associated kidney cancer (n = 16). *p* values from 2-sided Welch’s two sample *t*-test. (H) Bubble chart of expressions of marker genes for benign nephron cells in benign nephron cells and tumors; expressions of top ten marker genes from our normal cell atlas are shown (Figure S3 and Dataset S3). Abbreviations: BHD-ChRCC, BHD-associated chromophobe renal cell carcinoma; BHD-HOCT, BHD-associated hybrid oncocytic chromophobe tumor; ccFOXI1, confined cluster of *FOXI1* expressing cells; ccL1CAM, confined cluster of *L1CAM* expressing cells; ccRCC, clear cell renal cell carcinoma; cFOXI1, cluster of *FOXI1* expressing cells; cL1CAM, cluster of *L1CAM* expressing cells; HLRCC, HLRCC-associated kidney cancer; IC, intercalated cell; N, normal kidney; PC, principal cell; T, BHD-associated kidney cancer.

Recently, RNA *in situ* hybridization (RNA-ISH) revealed that BHD-associated HOCT was comprised of *L1 cell adhesion molecule* (*L1CAM*) expressing cells and *forkhead box I1* (*FOXI1*) expressing cells, whose expressions were mutually exclusive in each cell (Zhang et al., 2021b). Consistent with this earlier report, our single cell analysis of BHD-associated HOCT exhibited distinct clusters of *FOXI1* expressing cells and *L1CAM* expressing cells, supporting the notion that BHD-associated HOCT has transcriptomic intratumor heterogeneity (tITH) (Figure 4A). Notably, we found that a cluster of *L1CAM* expressing cells expresses marker genes for principal cells of collecting duct, whereas a cluster of *FOXI1* expressing cells and BHD-associated chromophobe renal cell carcinoma express marker genes for intercalated cells of collecting duct, suggesting that these distinctly clustered HOCT cells may develop in part by a mechanism similar to that of benign collecting duct cells (Figures 4B and S3A–S3D and Dataset S3). To further compare the molecular characteristics of collecting duct cells and HOCT cells, we conducted trajectory analysis and notably, principal cells were spotted at the initiation site of the trajectory. On the other hand, intercalated cells and a portion of the *L1CAM* expressing HOCT cells were scattered in the middle of the trajectory, and the *FOXI1* expressing HOCT cells were scattered at the terminus of the trajectory (Figures 4C and S4A–S4D). To uncover the underlying molecular mechanism, we investigated gene expressions and found that *FOXI1* as well as its downstream transcriptional target genes *ATPase H+ transporting V0 subunit a4* (*ATP6V0A4*), *ATPase H+ transporting V0 subunit d2* (*ATP6V0D2*), and *carbonic anhydrase 2 (CA2)* were upregulated in this trajectory (Figure 4D). *FOXI1* is an important transcription factor for intercalated cells that upregulates *ATP6V0A4* and *ATP6V0D2*, subunits of V-ATPase, an essential protein complex for acid-base homeostasis (Lindgren et al., 2017; Vidarsson et al., 2009). Indeed, we observed very strong expression of *FOXI1* and *FOXI1*-driven genes in the *FOXI1* expressing HOCT cells as well as moderate expression of *FOXI1* and *FOXI1*-driven genes even in the *L1CAM* expressing HOCT cells, suggesting that the elevated expressions of *FOXI1* and its downstream genes may be responsible for the intercalated cell characteristics in BHD-associated kidney cancer (Figures 4E–4G). In fact, expression levels of intercalated cell marker genes in BHD-associated kidney cancers exceeded their expression level in normal kidney, thereby supporting that the acquisition of intercalated cell characteristics in BHD-associated kidney cancer may be because of upregulation of *FOXI1* and its driven genes (Figures 4H and S3A–S3D and Dataset S3).

### Differentially expressed genes analysis highlights novel markers and a potential therapeutic target for BHD-associated kidney cancer

**Figure 5:**
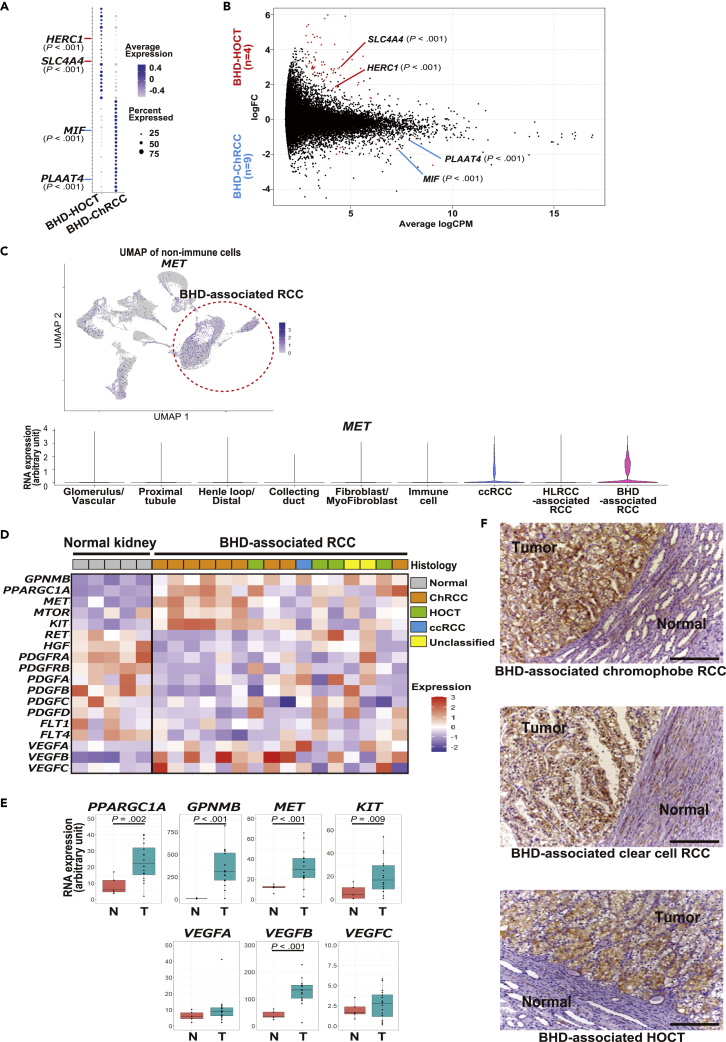
Differentially expressed genes (DEGs) analysis highlights novel markers and a potential therapeutic target for BHD-associated kidney cancer (A) Bubble chart of differentially expressed genes (DEGs) between BHD-associated HOCT (BHD-HOCT) and BHD-associated chromophobe renal cell carcinoma (BHD-ChRCC). Top twenty-five DEGs in each group are shown. (B) MA plot of DEGs between four BHD-HOCTs and nine BHD-ChRCCs bulk RNA-seq datasets. (C) UMAP plot of nonimmune cells from all of twelve specimens colored with *MET* expressions. The red dotted circle surrounds BHD-associated kidney cancer cells (upper panel). Violin plots of *MET* expressions in BHD-associated kidney cancers and other specimens (lower panel). *p* values from 2-sided Welch’s two sample *t*-test. (D) Heatmap of gene expressions in normal kidneys (n = 5) and BHD-associated kidney cancers (n = 16). (E) Boxplots of gene expressions shown in Figure 5D. N, normal kidney (n = 5). T, BHD-associated kidney cancer (n = 16). *p* values from 2-sided Welch’s two sample *t*-test. (F) Immunohistochemistry of MET protein in BHD-associated kidney cancers. Scale bars represent 200 μm. Abbreviations: BHD-ChRCC, BHD-associated chromophobe renal cell carcinoma; BHD-HOCT, BHD-associated hybrid oncocytic chromophobe tumor; ChRCC, chromophobe renal cell carcinoma; ccRCC, clear cell renal cell carcinoma; DEGs, differentially expressed genes; HOCT, hybrid oncocytic chromophobe tumor; N, normal kidney; T, BHD-associated kidney cancer; Unclassified, unclassified renal cell carcinoma.

To identify diagnostic markers distinguishing BHD-associated HOCT from BHD-associated chromophobe RCC, we investigated differentially expressed genes (DEGs) and found that while *HECT and RLD domain containing E3 ubiquitin protein ligase family member 1* (*HERC1*) (p < 0.001) and *solute carrier family 4 member 4* (*SLC4A4*) (p < 0.001) were highly expressed in BHD-associated HOCT, *macrophage migration inhibitory factor* (*MIF*) (p < 0.001) and *phospholipase A and acyltransferase 4* (*PLAAT4*) (p < 0.001) were highly expressed in BHD-associated chromophobe RCC, suggesting that these genes might be novel diagnostic marker genes to distinguish BHD-associated HOCT from BHD-associated chromophobe renal cell carcinoma (Figures 5A and 5B).

To search for a molecular therapeutic target for BHD-associated kidney cancer, we compared DEGs identified in BHD-associated kidney cancers with DEGs in other cancers analyzed in this study, and found that *MET* was highly expressed in BHD-associated kidney cancers (Figure 5C). Bulk RNA-seq datasets from 16 BHD-associated kidney cancers and 5 normal kidneys further showed that the expression levels of *MET* (p < 0.001) and *vascular endothelial growth factor B* (*VEGFB*) (p < 0.001) in BHD-associated kidney cancers were statistically higher than those in normal kidneys (Figures 5D and 5E and S4E and S4F). Finally, using anti-MET immunohistochemistry, we confirmed that MET protein expression was greater in BHD-associated kidney cancers than in adjacent normal kidney tissue (Figure 5F).

## Discussion

In this study, we compared the single-cell transcriptomes of primary tumors of hereditary kidney cancers and the cells that make up the tumor microenvironment and uncovered unique characteristics of tumor cells and microenvironment cells for each hereditary kidney cancer at single-cell level. For instance, HLRCC-associated kidney cancer harbors suppressed T cells and *TREM2*-high macrophages, which may lead to immune tolerance. BHD-associated kidney cancer is an indolent tumor as shown in Figure 3B. In addition, we observed transcriptomic intratumor heterogeneity (tITH) and increased intercalated cell characteristics with upregulated *FOXI1*-driven genes in BHD-associated kidney cancer. The results presented in this study uncover how tumor cells modulate gene expressions to adapt to their tissue microenvironments (Gupta and Massague, 2006).

On a molecular basis, HLRCC-associated kidney cancer has been reported to exhibit a CpG island methylator phenotype (CIMP) in which gene expressions are extensively suppressed because of hypermethylation of CpG islands (Cancer Genome Atlas Research et al., 2016). Dysregulation of *fumarate hydratase (FH)*, the causative gene for HLRCC, results in the accumulation of fumarate and inhibition of alpha ketoglutarate-dependent hydroxylases that catalyze DNA demethylation (Hasumi and Yao, 2018). In this study, HLRCC-associated kidney cancer formed a more distant cluster from nephron cells compared with other types of kidney cancers, indicating that extensive transcriptional suppression because of CIMP may affect gene expressions that support nephron cell characteristics in HLRCC-associated kidney cancer. Interestingly, although no additional driver gene alteration was detected by whole exome sequencing of the HLRCC-associated lymph node metastasis as compared to the primary lesion, the primary lesion and lymph node metastasis clustered independently. The lymph node metastasis completely lost expression of *SLC17A3*, a proximal tubule marker, suggesting that the hypermethylation of CpG islands in HLRCC-associated kidney cancer may further affect gene expressions to adapt to the lymph node tissue microenvironment. Therefore, it will be interesting to compare the CIMP status of the lymph node metastasis and primary lesion to determine if CpG island hypermethylation in HLRCC-associated kidney cancer may trigger the modulation of gene expressions and promote adaptation of tumor cells to the lymph node tissue microenvironment.

BHD-associated HOCT exhibited transcriptomic intratumor heterogeneity (tITH) comprised of *L1CAM* and *FOXI1* expressing cells, suggesting the possibility that the origin cell of BHD-associated HOCT may have already been genetically altered before differentiating into these two transcriptomically distinct lineages. Intriguingly, trajectory analysis revealed that principal cells were plotted at the initiation site of the trajectory, and *FOXI1* and its downstream genes — *ATP6V0A4*, *ATP6V0D2,* and *carbonic anhydrase 2 (CA2)* — were upregulated in this trajectory. *FOXI1* is a known transcription factor for collecting duct differentiation. Recently, an association between *FOXI1* and the mTOR pathway was identified. Mice with principal cell-specific inactivation of *TSC complex subunit* (*Tsc1*), a modulator of mTOR kinase activity, develop renal cysts composed of hyperproliferative intercalated cells with robust expressions of *Foxi1* and its downstream genes *Ca2* and subunits of V-ATPase that are critical components of intercalated cells. This phenotype was completely abrogated by the inactivation of *Foxi1*, suggesting that *FOXI1* may act as a critical mediator for the TSC-mTOR pathway through its regulation of collecting duct differentiation (Barone et al., 2021). *FLCN*, a causative gene for BHD syndrome, is also associated with the mTOR pathway by signaling amino acid levels to mTORC1 (Tsun et al., 2013). Notably, in this study we observed increased expression of *FOXI1* in BHD-associated kidney cancers. These findings suggest that BHD-associated kidney cancer may acquire characteristics of intercalated cells with upregulated *FOXI1*-driven genes following genomic alteration of its cell of origin.

To date, there is no effective form of therapy for BHD-associated kidney cancer. In our study, the number of cytotoxic T cells in BHD-associated kidney cancer is quite small and HIF-VEGF signaling is not upregulated in BHD-associated vasculature, suggesting that neither immune checkpoint inhibitors nor angiogenesis inhibitors may be effective against this cancer; therefore, novel therapeutic approaches are needed based on the molecular context of BHD-associated kidney cancer. In the present study, we found that *MET* expression was high in various histological types of BHD-associated kidney cancer, raising the possibility that MET inhibitors, such as cabozantinib and crizotinib, may provide promising therapeutic approaches for treatment of BHD-associated kidney cancer.

Although we conducted an analysis of transcriptomic intratumor heterogeneity (tITH) by analyzing three spatially distant lesions in s-ccRCC2, we did not find any differences in transcriptomes from these three lesions (Figures S5A–S5C). As we have shown in Figure 1B, we observed genomic intratumor heterogeneity (gITH) in these three lesions with a variety of driver gene variants including a *retinoblastoma 1* (*RB1*) variant, but only *VHL* and *lysine demethylase 5C* (*KDM5C*) were commonly altered in all three lesions, suggesting that *VHL* and *KDM5C* may be founder variants for these three distant lesions. Therefore, the transcriptomes of tumor cells and infiltrating immune cells may be largely driven by founder variants in kidney cancer associated genes such as *VHL* and *KDM5C*.

Our single-cell transcriptome analysis of various types of hereditary kidney cancers illustrates how tumor cells associated with a deficiency of each of the causative kidney cancer-associated genes modulate gene expressions and adapt to their tissue microenvironment. Molecular characteristics of tumor cells and the tissue microenvironment revealed in this study provide an opportunity for the development of novel therapeutics that target tumor cells and the tissue microenvironment, which are specific to genetic alterations associated with each kidney cancer associated gene.

### Limitations of the study

Because cancer cell characteristics are largely affected by the genetic background of cancer cells, we decided to analyze hereditary kidney cancer in this study to minimize the effect of genetic variations. However, hereditary kidney cancer might still have genetic alterations in addition to the loss of its responsible gene, which may affect tumor characteristics. Because tissue microenvironment may vary between individuals, a large-scaled single cell RNA-seq study is necessary to eliminate variations between individuals. Molecular characteristics of cancer cells may be sometimes different from that of a cell of origin. Thus, we need to carefully pursue cells of origin of kidney cancers using multiple methodologies.

## STAR★Methods

### Key resource table

**Table:** 

REAGENT or RESOURCE	SOURCE	IDENTIFIER
**Antibodies**		

Rabbit monoclonal anti-MET (clone D1C2)	Cell Signaling Technology	Cat# 8198S, RRID:AB_10858224
Dako REAL EnVision Detection System, Peroxidase/DAB, Rabbit/Mouse, HRP Kit	Agilent	Cat# K5007, RRID:AB_2888627

**Chemicals, peptides, and recombinant proteins**		

Liberase DL	Sigma-Aldrich	Cat# 05,466,202,001
DNase I	Sigma-Aldrich	Cat# 10104159001
RBC lysis buffer	BD Biosciences	Cat# 555899

**Critical commercial assays**		

Cellometer Auto 2000 Cell Viability Counter	Nexcelom Bioscience	N/A
Chromium Single Cell Controller instrument	10× Genomics	N/A
Chromium Next GEM Chip G Single Cell Kit	10× Genomics	Cat# 1000127
Chromium Single Cell 3ʹ GEM, Library & Gel Bead Kit v3.1	10× Genomics	Cat# 1000128
Chromium i7 Multiplex Kits	10× Genomics	Cat# 120262
NextSeq 500/550 High Output Kit v2.5	Illumina	Cat# 20024907
KAPA Library Quantification Kit	Roche	Cat# KK4828

**Deposited data**		

scRNA sequencing data	This study	DDBJ:JGAS000303
RNA sequencing data	This study	DDBJ:JGAS000303
Whole exome sequencing data	This study	DDBJ:JGAS000303
TCGA KIRC RNA sequencing data	GDC Data Portal	https://portal.gdc.cancer.gov/

**Software and algorithms**		

Seurat (v3.1.2)	Stuart et al. (2019)	https://satijalab.org/seurat/
RStudio (v3.6.3)	RStudio, Inc.	https://www.rstudio.com/products/rstudio/
CIBERSORTx	Newman et al. (2019)	https://cibersortx.stanford.edu/
survival (v3.2.13)	Therneau T. M., 2020	https://CRAN.R-project.org/package=survival
Cell Ranger (v6.0.2)	10× Genomics	https://support.10xgenomics.com/
monocle2 (v2.14.0)	Qiu et al. (2017)	http://cole-trapnell-lab.github.io/monocle-release/
Cytoscape (v3.7.2)	Shannon et al. (2003)	https://cytoscape.org/
fgsea (v1.16.0)	Innis et al. (2021)	https://github.com/ctlab/fgsea
Genomon (v2.6.3)	the Human Genome Center (HGC) supercomputer system at The University of Tokyo	https://github.com/Genomon/genomon
Riboduct	N/A	https://github.com/msfuji/riboduct

### Resource availability

#### Lead contact

Further information and requests for resources and reagents should be directed to and will be fulfilled by the lead contact, Hisashi Hasumi (hasumi@yokohama-cu.ac.jp).

#### Materials availability

This study did not generate new unique reagents.

### Experimental model and subject details

#### Patients and specimens

Patients known or suspected to be affected with hereditary or sporadic kidney cancers were referred to the Department of Urology in Yokohama City University Hospital, which is a center for hereditary kidney cancer in Japan. Germline alteration of each hereditary kidney cancer patient was confirmed by Sanger sequencing prior to surgery; one BHD patient with chromophobe renal cell carcinoma (BHD-ChRCC) was found to have a germline variant of c.404delC in *folliculin* (*FLCN*) gene, another BHD patient with hybrid oncocytic chromophobe tumor (BHD-HOCT) had a germline variant of c.1533–1536 delGATG in *FLCN* gene, HLRCC patient had a germline variant of c.712G > C in *fumarate hydratase (FH)* gene resulting in amino acid change of Asp238His in the FH protein, one VHL patient had a large deletion encompassing exons 1 and 2 of *VHL* gene at germline level and another VHL patient had a germline variant of c.226–228 delTCT in *VHL* gene (Figures S5D–S5H). VHL1 patient developed bilateral multifocal clear cell renal cell carcinomas metachronously, which is a characteristic of VHL-associated kidney cancer. The primary lesion of HLRCC-associated kidney cancer and its lymph node metastasis were resected in the same operation. Germline variant of *FH* pSer334Arg in exon 7 was identified in s-ccRCC1 patient, who is a family member of an HLRCC kindred, and a germline *FLCN* c.199 dupG variant was coincidentally identified in s-ccRCC2 patient, without any somatic second-hit variant being detected in these genes; therefore, both s-ccRCC1 and s-ccRCC2 were categorized as sporadic kidney cancers in this study. Two normal kidney samples were obtained from normal renal cortexes of s-ccRCC1 and s-ccRCC2 patients. To analyze intratumor heterogeneity, s-ccRCC2 was sampled three times and each sample was named as s-ccRCC2-1, s-ccRCC2-2 and s-ccRCC2-3, respectively. Either robotic partial nephrectomy or open nephrectomy was performed to remove the tumors in these patients.

#### TCGA KIRC data

Bulk RNA sequencing data of 539 tumors and patient’s clinical information in TCGA project were downloaded from the GDC data repository (https://portal.gdc.cancer.gov/).

#### Ethics statement

This study was approved by the Institutional Review Board of Yokohama City University (A200100004), which does not permit to disclose any personal information of patient. Informed consent for the study was obtained from all participants.

### Method details

#### Single-cell RNA sequencing

Single-cell RNA sequencing was performed on twelve surgically resected specimens from seven patients including two BHD-associated kidney cancers, one primary lesion and one lymph node metastasis from HLRCC-associated kidney cancer, two VHL-associated kidney cancers, one sporadic clear cell renal cell carcinoma (ccRCC), 3 independent samplings from a second ccRCC and two normal kidney tissues. Surgically resected specimens were sliced into approximately 50 mm^3^ sized pieces, minced and digested in 1.5 mL of 1× PBS supplemented with 0.33 mg/mL Liberase DL (Sigma-Aldrich) and 0.2 mg/mL DNase at 37 °C for 30 min. After adding 150 μL of 100% FBS, each cell suspension was passed through a 40 μm cells strainer and cells were collected by centrifugation of 1000 rpm for 5 min at 4 °C. Red blood cells were removed by incubating with RBC lysis buffer (BD Biosciences, Franklin Lakes, NJ) for 3 min on ice, following centrifugation at 1000 rpm for 5 min at 4 °C. Cell pellet was resuspended with 1 mL of 1× PBS supplemented with 0.5% BSA. Live cells were counted by Cellometer Auto (2000) (Nexcelom Bioscience). Single cell suspensions were loaded on a Chromium Single Cell Controller instrument (10× Genomics) to generate emulsion droplets containing cells and beads. Single-cell RNA-Seq libraries were prepared using the Chromium single cell 3′ kit (10× Genomics) according to the manufacturer’s protocol. The library quantification was performed using KAPA Library Quantification Kit (Roche). Paired-end sequencing was conducted on the NextSeq 500 platform (Illumina). Read alignment and quantification were done using Cell Ranger (6.0.2) and the GRCh38 reference genome (Dataset S4). The Quality Control (QC) process was done using R package Seurat (version 3.1.2) (Stuart et al., 2019). Single cells with less than 200 UMIs were removed as low-quality cells. We chose genes for analysis that were expressed in equal or more than 3 cells. We normalized our data using NormalizeData function in Seurat (scale.factor = 10000). We identified highly variable features using FindVariableFeatures function with top 2,000 variable genes. Data from twelve specimens were integrated using the IntegrateData and FindIntegrationAnchors functions. We applied a linear transformation using the ScaleData function. Principal component analysis (PCA) was done using the RunPCA function. We determined the dimension of the dataset using the ElbowPlot function and we adopted optimal PC. We annotated cell clusters using the FindClusters function (resolution = 0.5–1.2) and mapped them into 2D uniform manifold approximation and projection (UMAP). 108,342 single cells, including 11,839 normal specimens-derived cells, 89,573 primary tumor specimens-derived cells and 6,930 lymph node metastasis specimen-derived cells were subjected to further analyses. Pseudotime trajectory analysis was performed using R package monocle2 (version 2.14.0) (Qiu et al., 2017).

#### Deconvolution of bulk RNA-seq datasets

Deconvolution of bulk RNA-seq datasets was done using an analytical tool CIBERSORTx (https://cibersortx.stanford.edu/) (Newman et al., 2019). First, a reference matrix for each cell type was created using the single-cell RNA sequencing data of clear cell RCC samples analyzed in this study, and then the percentage of component cells in clear cell RCC samples in TCGA KIRC project was calculated by deconvolution methods in CIBERSORTx using the reference matrix. Bulk RNA sequencing data of 539 tumors in TCGA KIRC project were obtained from the GDC data portal (https://portal.gdc.cancer.gov/). Default parameters were applied; 999 for Maximum condition number (kappa) for signature matrix, 0.01 for *q* value cutoff for differential expression analysis and 300 and 500 for minimum and maximum number of barcode genes to consider for each phenotype when building signature matrix, respectively. Signature matrices and lists of cell fractions are available in Figures S2A–S2D and Dataset S2.

#### Patient survival analysis based on cell type composition

Overall survival in patients from TCGA KIRC project was predicted based on cell type composition. Patients were divided into two groups using median ratio of cell number of each cell type (cancer cell, vascular cell, TAM, cytotoxic T cell or B cell) per total cell number. Patient’s clinical information in TCGA KIRC project was obtained from the GDC data portal (https://portal.gdc.cancer.gov/). Survival analysis was performed using Kaplan-Meier survival curves. *p* values from 2-sided log rank test, and the hazard ratio with 95% confidence interval is calculated.

#### Gene ontology (GO) analysis and gene set enrichment analysis (GSEA)

GO analyses were done using Cytoscape software (version 3.7.2) plug-in BiNGO; the cutoff threshold for p value was set as 0.05 (Shannon et al., 2003). GSEA were done using R package fgsea (version 1.16.0); default parameters were applied; 10,000 for nperm, 15 for minSize and 1000 for maxSize (Innis et al., 2021).

#### Whole exome sequencing

Whole exome sequencing was done using SureSelect Human All Exon V6 (Agilent Technologies) and Illumina Novaseq6000/PE150. Reads mapping and further analyses were done using Genomon pipeline (https://github.com/Genomon/genomon). The GRCh37 reference genome was used for alignment.

#### RNA sequencing of bulk tissues

RNA sequencing was done with bulk tissues of 16 BHD-associated kidney cancers and 5 adjacent normal kidneys from ten BHD patients using TruSeq Stranded mRNA Library Prep Kit, TruSeq RNA CD Index Kit (Dual Index) and Hiseq 2500 (Illumina) according to the manufacturer protocol. Reads mapping and further analyses were done using GitHub RNA-seq pipeline riboduct (https://github.com/msfuji/riboduct). The GRCh37 reference genome was used for alignment.

#### Immunohistochemistry

Immunohistochemistry was done on 4-μm-thick representative formalin-fixed paraffin-embedded sections. The tissue slides were autoclaved in Tris-EDTA buffer (pH 9.0) for antigen retrieval, then incubated with rabbit monoclonal MET antibody at 1:300 dilution (clone D1C2, #8198; Cell Signaling Technology) for one hour at room temperature and visualized with the Real EnVision Detection System, Peroxidase/DAB+, Rabbit/Mouse (Agilent Technologies). The MET protein was considered to be positive when membranous and/or cytoplasmic MET staining was observed.

### Quantification and statistical analysis

Statistical analyses were performed using Rstudio (version 3.6.3). Welch’s two sample t-test was applied to determine whether the means of two populations were different, and differences were considered to be statistically significant at a value of p < 0.05. All statistical tests were 2-sided. The association between cell type compositions of clear cell renal carcinomas and patients’ outcomes were analyzed with the Kaplan-Meier method and 2-sided log rank test.

## Data Availability

• All of sequencing data, including scRNA, RNA and whole exome sequencing, were deposited in DNA DataBank of Japan (DDBJ) under the accession number JGAS000303. Raw quantification data for each figure were deposited at online repository in Mendeley (https://data.mendeley.com/drafts/7w3x9kfnbm). • All codes used in this study were from already existing software and algorithms, as listed in the “Software and Algorithms” section of the key resource table. • Any additional information required to reanalyze the data reported in this work paper is available from the lead contact upon request.

## Supplemental information

Document S1. Figures S1–S5

Dataset S1. Markers of nonimmune cell clusters from 12 samples, related to Figure 1

Dataset S2. Signature matrix for deconvolution method, related to Figure 2

Dataset S3. Markers of nonimmune cell clusters from normal cell atlas, related to Figure 4

Dataset S4. Summary of single cell sequencing quality, related to STAR Methods
