# Supplementary material for: Single-cell transcriptomes underscore genetically distinct tumor characteristics and microenvironment for hereditary kidney cancers
Source: iScience. 2022 May 25;25(6):104463. doi: 10.1016/j.isci.2022.104463 (PMC9301876; doi:10.1016/j.isci.2022.104463)
Supplement: Document S1. Figures S1–S5 [file mmc1.pdf]

iScience, Volume *25*

Supplemental information

Single-cell transcriptomes underscore genetically distinct tumor characteristics and microenvironment for hereditary kidney cancers

Ryosuke Jikuya, Koichi Murakami, Akira Nishiyama, Ikuma Kato, Mitsuko Furuya, Jun Nakabayashi, Jordan A. Ramilowski, Haruka Hamanoue, Kazuhiro Maejima, Masashi Fujita, Taku Mitome, Shinji Ohtake, Go Noguchi, Sachi Kawaura, Hisakazu Odaka, Takashi Kawahara, Mitsuru Komeya, Risa Shinoki, Daiki Ueno, Hiroki Ito, Yusuke Ito, Kentaro Muraoka, Narihiko Hayashi, Keiichi Kondo, Noboru Nakaigawa, Koji Hatano, Masaya Baba, Toshio Suda, Tatsuhiko Kodama, Satoshi Fujii, Kazuhide Makiyama, Masahiro Yao, Brian M. Shuch, Laura S. Schmidt, W. Marston Linehan, Hidewaki Nakagawa, Tomohiko Tamura, and Hisashi Hasumi

Figure S1

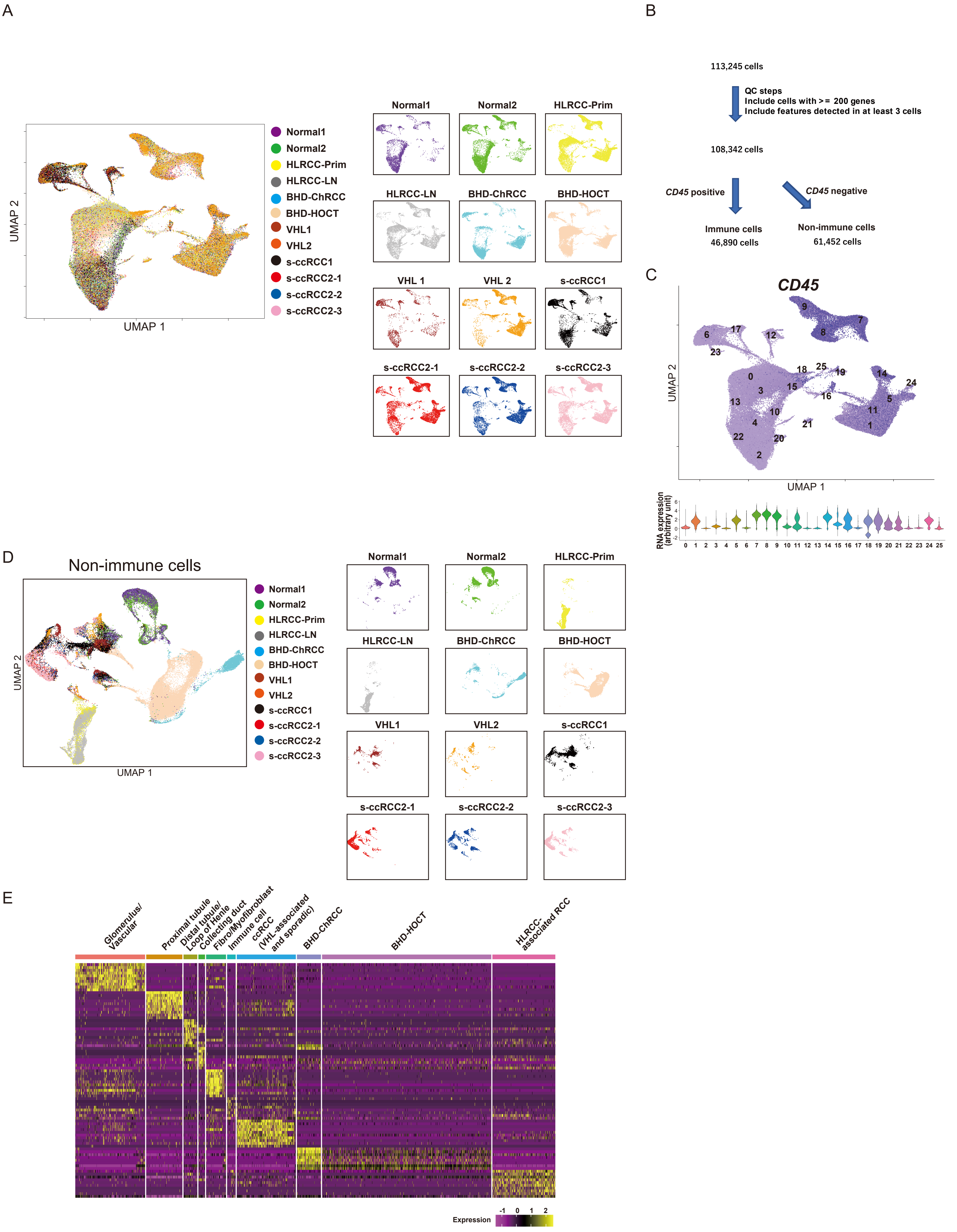
A
B
UMAP 2
UMAP 1
Normal1
Normal2
HLRCC-Prim
HLRCC-LN
BHD-ChRCC
BHD-HOCT
VHL1
VHL2
s-ccRCC1
s-ccRCC2-1
s-ccRCC2-2
s-ccRCC2-3
VHL 1
VHL 2
113,245 cells
QC steps
Include cells with >= 200 genes
Include features detected in at least 3 cells
108,342 cells
CD45 positive
CD45 negative
Immune cells
46,890 cells
Non-immune cells
61,452 cells
C
CD45
RNA expression (arbitrary unit)
D
Non-immune cells
E
Glomerulus/ Vascular
Proximal tubule
Distal tubule/ Loop of Henle
Collecting duct
Fibro/Myofibroblast
Immune cell
ccRCC (VHL-associated and sporadic)
BHD-ChRCC
BHD-HOCT
HLRCC-associated RCC
Expression

Figure S1

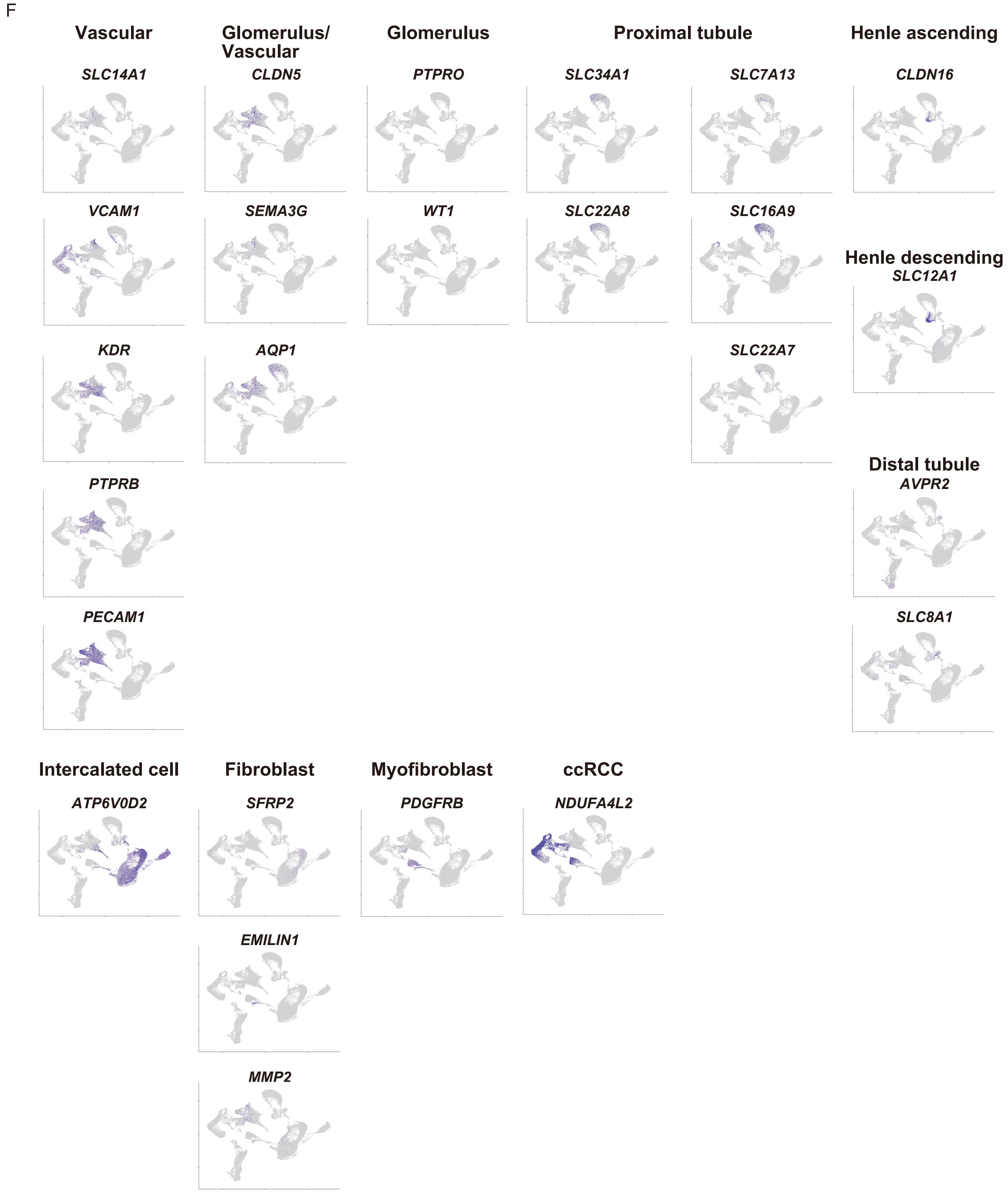
F
Vascular
SLC14A1
VCAM1
KDR
PTPRB
PECAM1
Glomerulus/ Vascular
CLDN5
SEMA3G
AQP1
Glomerulus
PTPRO
WT1
Proximal tubule
SLC34A1
SLC22A8
SLC7A13
SLC16A9
SLC22A7
Henle ascending
CLDN16
Henle descending
SLC12A1
Distal tubule
AVPR2
SLC8A1
Intercalated cell
ATP6V0D2
Fibroblast
SFRP2
EMILIN1
MMP2
Myofibroblast
PDGFRB
ccRCC
NDUFA4L2

Figure S1

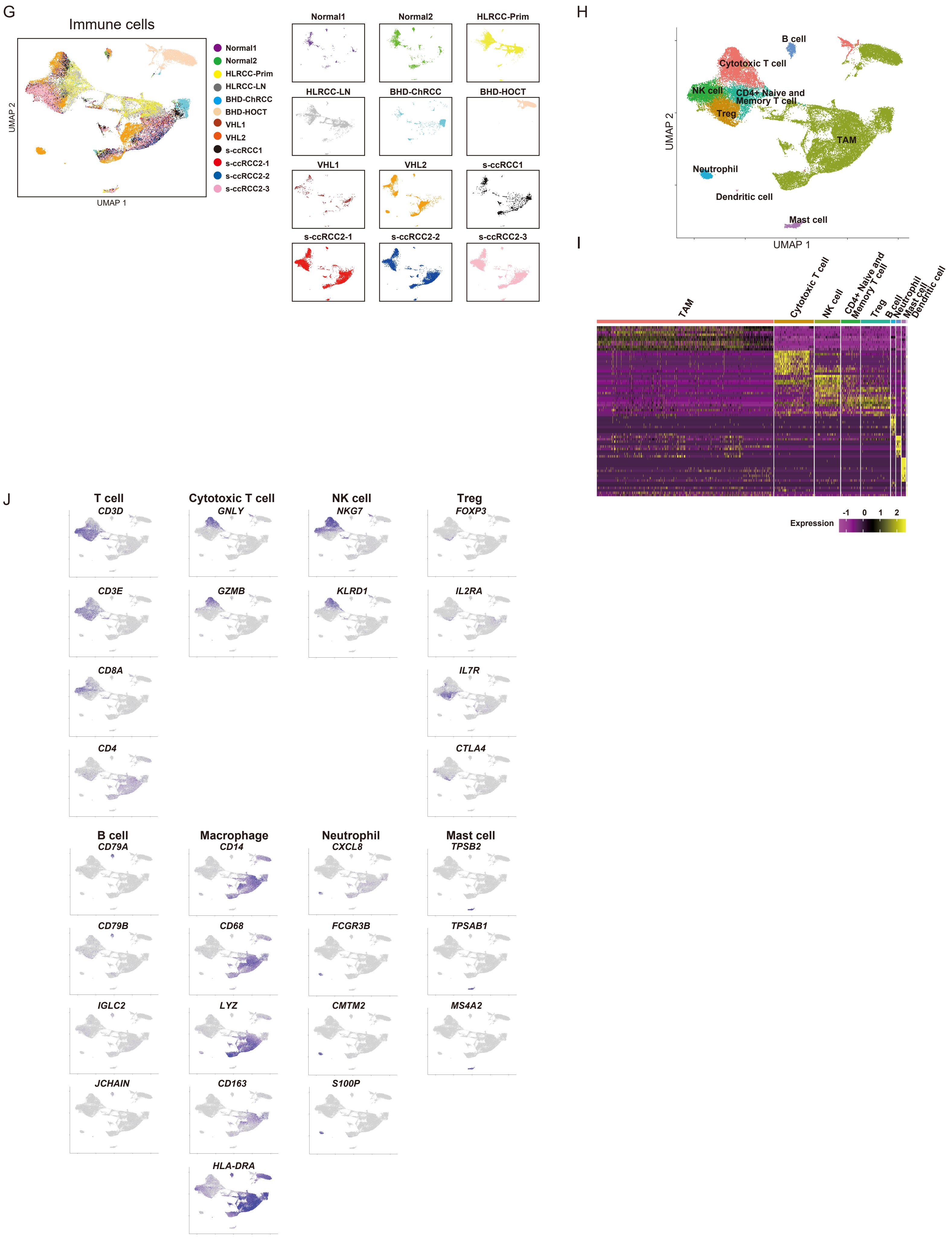
G
Immune cells
UMAP 2
UMAP 1
Normal1
Normal2
HLRCC-Prim
HLRCC-LN
BHD-ChRCC
BHD-HOCT
VHL1
VHL2
s-ccRCC1
s-ccRCC2-1
s-ccRCC2-2
s-ccRCC2-3
H
B cell
Cytotoxic T cell
NK cell
CD4+ Naive and Memory T cell
Treg
TAM
Neutrophil
Dendritic cell
Mast cell
UMAP 2
UMAP 1
I
TAM
Cytotoxic T cell
NK cell
CD4+ Naive and Memory T cell
Treg
B cell
Neutrophil
Mast cell
Dendritic cell
Expression
-1 0 1 2
J
T cell
CD3D
CD3E
CD8A
CD4
Cytotoxic T cell
GNLY
GZMB
NK cell
NKG7
KLRD1
Treg
FOXP3
IL2RA
IL7R
CTLA4
B cell
CD79A
CD79B
IGLC2
JCHAIN
Macrophage
CD14
CD68
LYZ
CD163
HLA-DRA
Neutrophil
CXCL8
FCGR3B
CMTM2
S100P
Mast cell
TPSB2
TPSAB1
MS4A2

Figure S1. Overview of quality control steps for creation of cell atlas from all of 12 samples and annotation of non-immune or immune cells. Related to Figure 1. (A) UMAP plots show 108,342 cells from 12 specimens analyzed in this study. (B) Overview of quality control and cell allocation steps. (C) *CD45*, an immune cell marker, was used to divide total 108,342 cells into 46,890 immune cells and 61,452 non-immune cells. The number on the UMAP and in the violin plot indicates a cluster number automatically assigned by Seurat software. (D) UMAP plots show *CD45* negative non-immune cells in each specimen. (E) Heatmap shows gene expressions in annotated clusters in Figure 1E. (F) UMAP plots show marker genes expressions in non-immune cells, which were used for the cell annotation. Some of them were shown in Figure 1E. (G) UMAP plots show *CD45* positive immune cells in each specimen. (H) UMAP plot shows *CD45* positive immune cells from all of 12 specimens annotated with marker genes. (I) Heatmap shows gene expressions in annotated clusters. (J) UMAP plots show marker genes expression in immune cells, which were used for the cell annotation. Abbreviations: BHD-ChRCC, BHD-associated chromophobe renal cell carcinoma; BHD-HOCT, BHD-associated hybrid oncocytic chromophobe tumor; ccRCC, clear cell renal cell carcinoma; Henle ascending, ascending limb of the loop of Henle; Henle descending, descending limb of the loop of Henle; HLRCC-LN, Lymph node metastasis of HLRCC-associated kidney cancer; HLRCC-Prim, Primary lesion of HLRCC-associated kidney cancer; NK cell, Natural killer cell; s-ccRCC, sporadic clear cell renal cell carcinoma; TAM, tumor associated macrophage; Treg, regulatory T cell.

Figure S2

A

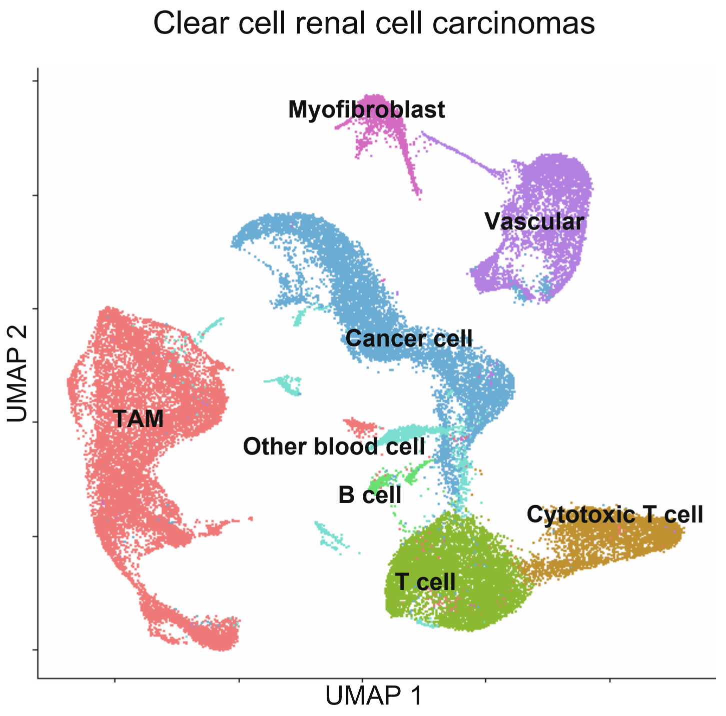

B

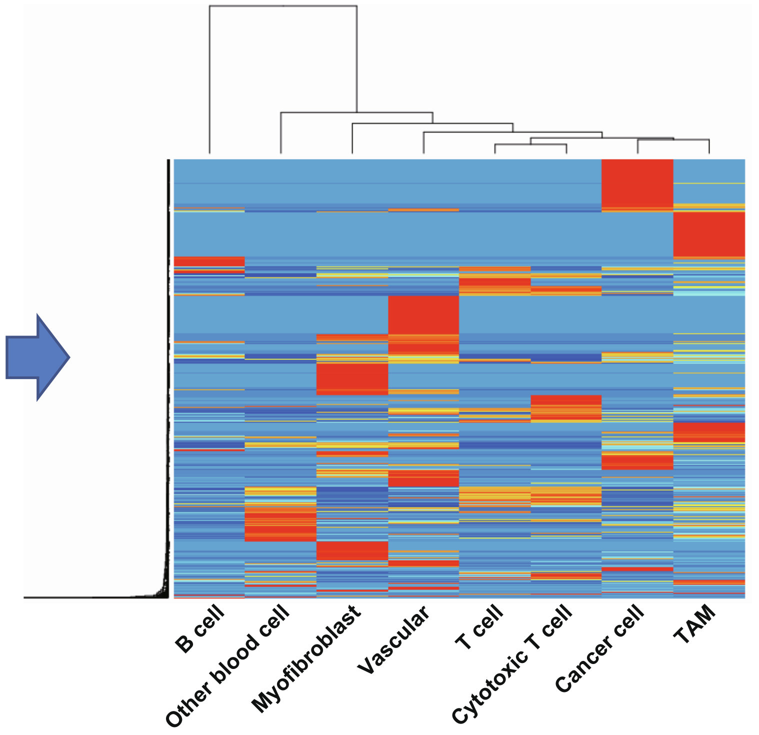

C

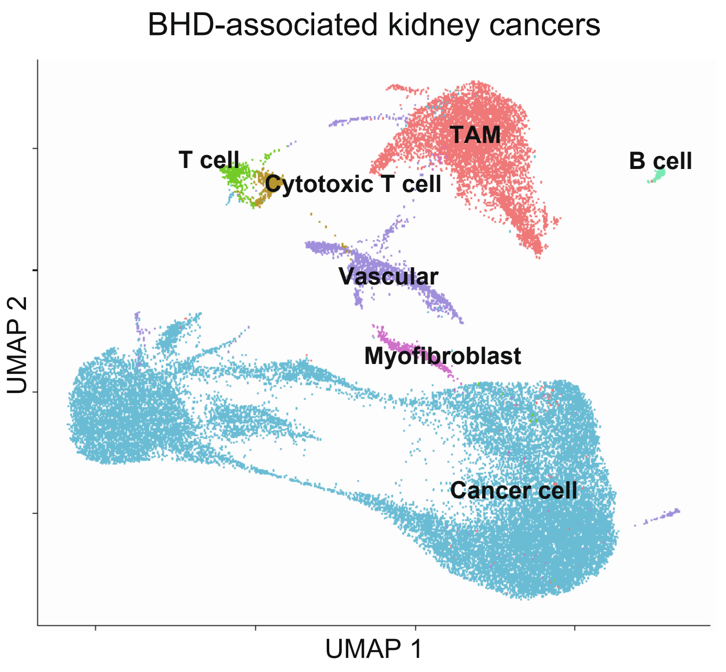

D

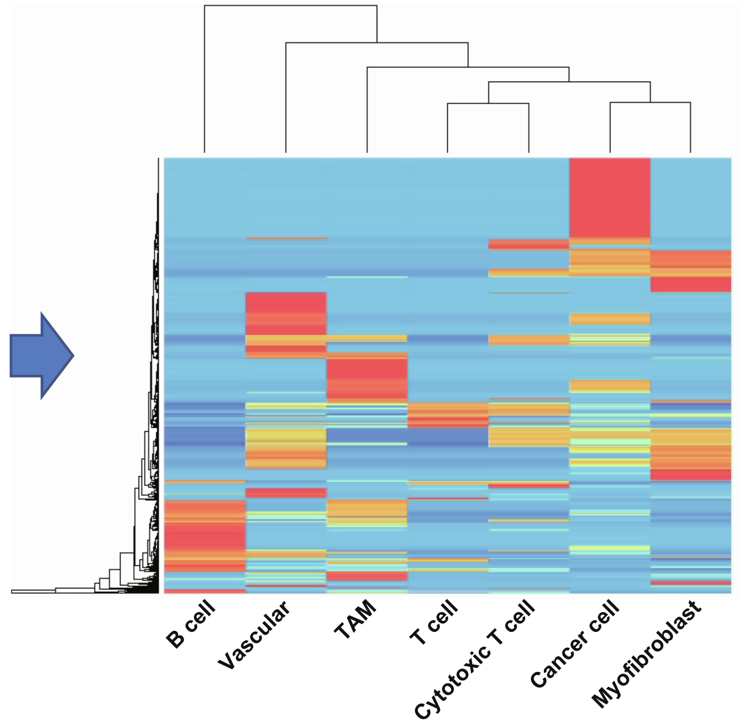

Figure S2

E

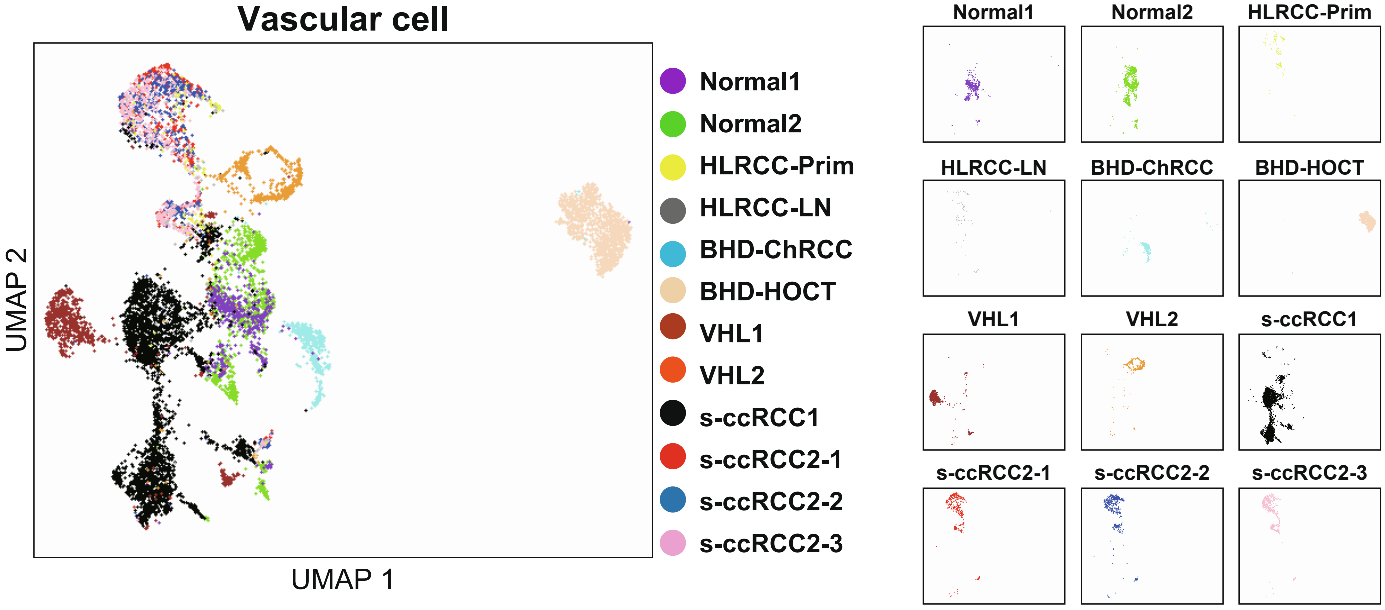

F

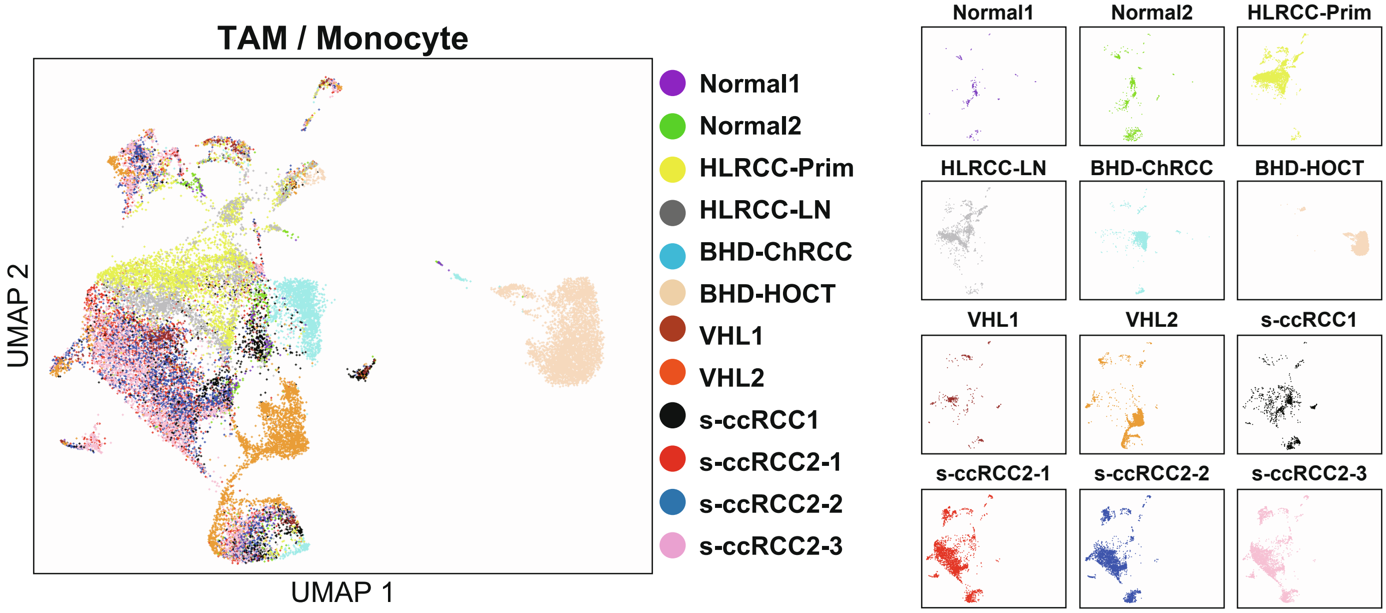

G

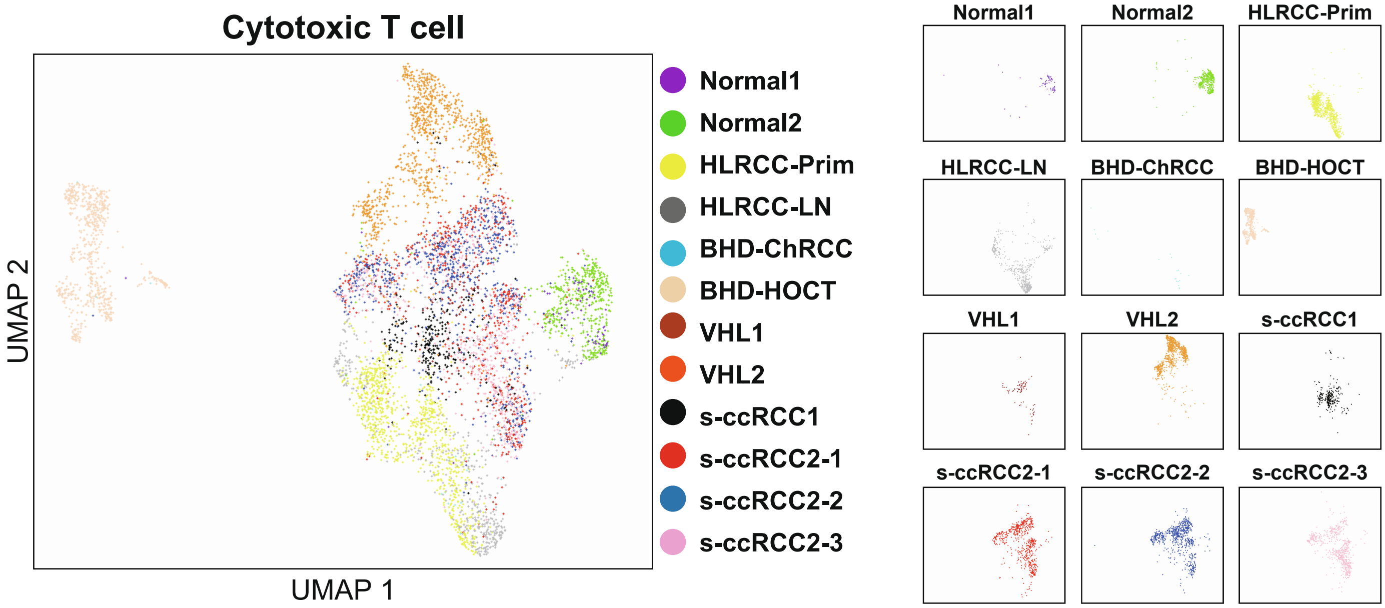

Figure S2. Reference matrices and signature matrices for cell type composition analyses of deconvolution pipeline and clustering of tumor microenvironment cells. Related to Figure 2. (A) UMAP plot shows 42,406 cells from six clear cell renal cell carcinomas (two VHL-associated and four sporadic). (B) Heatmap shows gene expressions in each cluster (Signature matrix). (C) UMAP plot shows 35,371 cells from two BHD-associated kidney cancers. (D) Heatmap shows gene expressions in each cluster (Signature matrix). (E) UMAP plots show vascular cells in each specimen. (F) UMAP plots show tumor associated macrophages (TAM) or monocytes in each specimen. (G) UMAP plots show cytotoxic T cells in each specimen. Abbreviations: BHD-ChRCC, BHD-associated chromophobe renal cell carcinoma; BHD-HOCT, BHD-associated hybrid oncocytic chromophobe tumor; HLRCC-LN, Lymph node metastasis of HLRCC-associated kidney cancer; HLRCC-Prim, Primary lesion of HLRCC-associated kidney cancer; s-ccRCC, sporadic clear cell renal cell carcinoma; TAM, tumor associated macrophage.

Figure S3

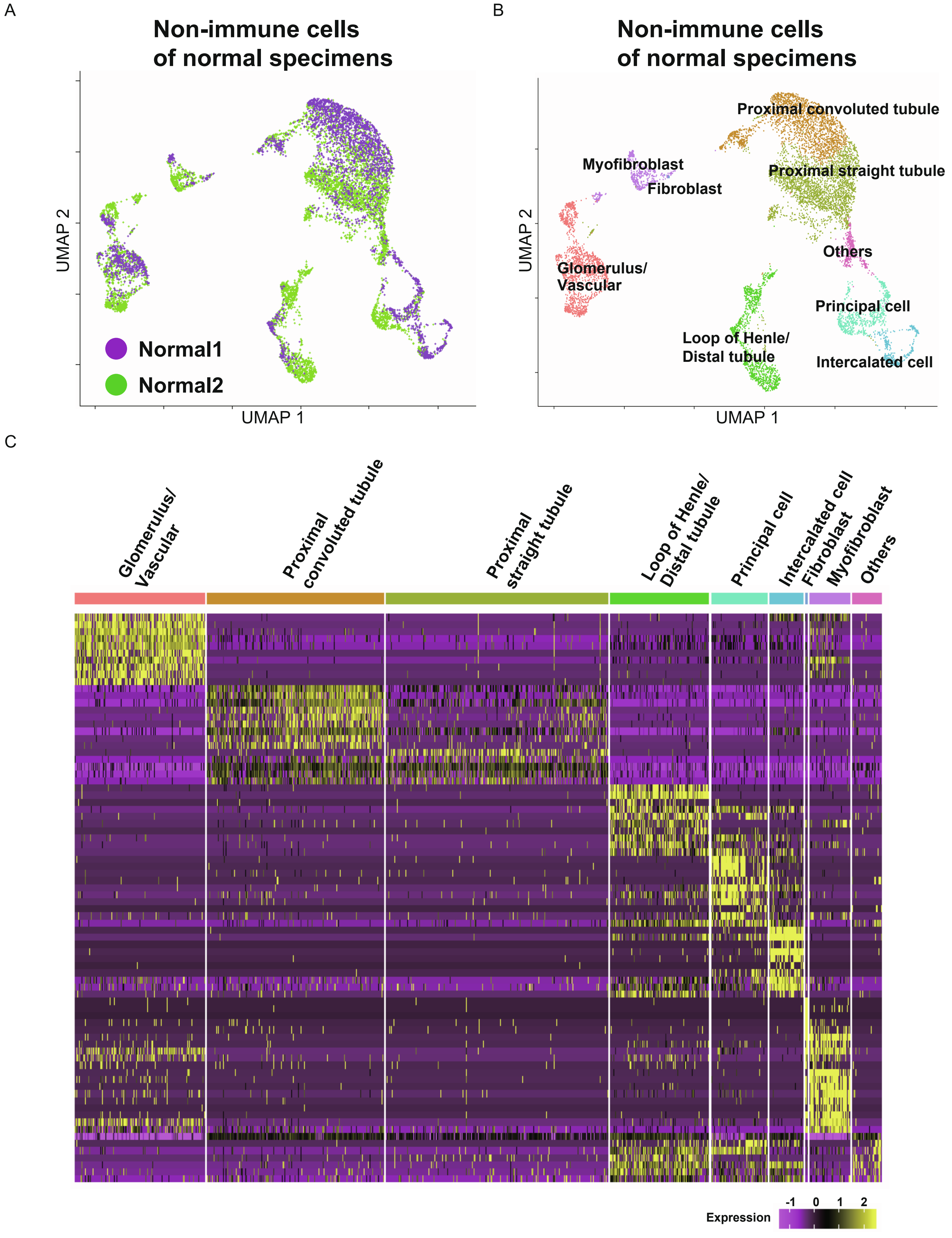

A
Non-immune cells of normal specimens
UMAP 2
UMAP 1
Normal1
Normal2
B
Non-immune cells of normal specimens
Proximal convoluted tubule
Myofibroblast
Fibroblast
Proximal straight tubule
Others
Glomerulus/ Vascular
Principal cell
Loop of Henle/ Distal tubule
Intercalated cell
UMAP 2
UMAP 1
C
Glomerulus/ Vascular
Proximal convoluted tubule
Proximal straight tubule
Loop of Henle/ Distal tubule
Principal cell
Intercalated cell
Fibroblast
Myofibroblast
Others
Expression
-1
0
1
2

Figure S3

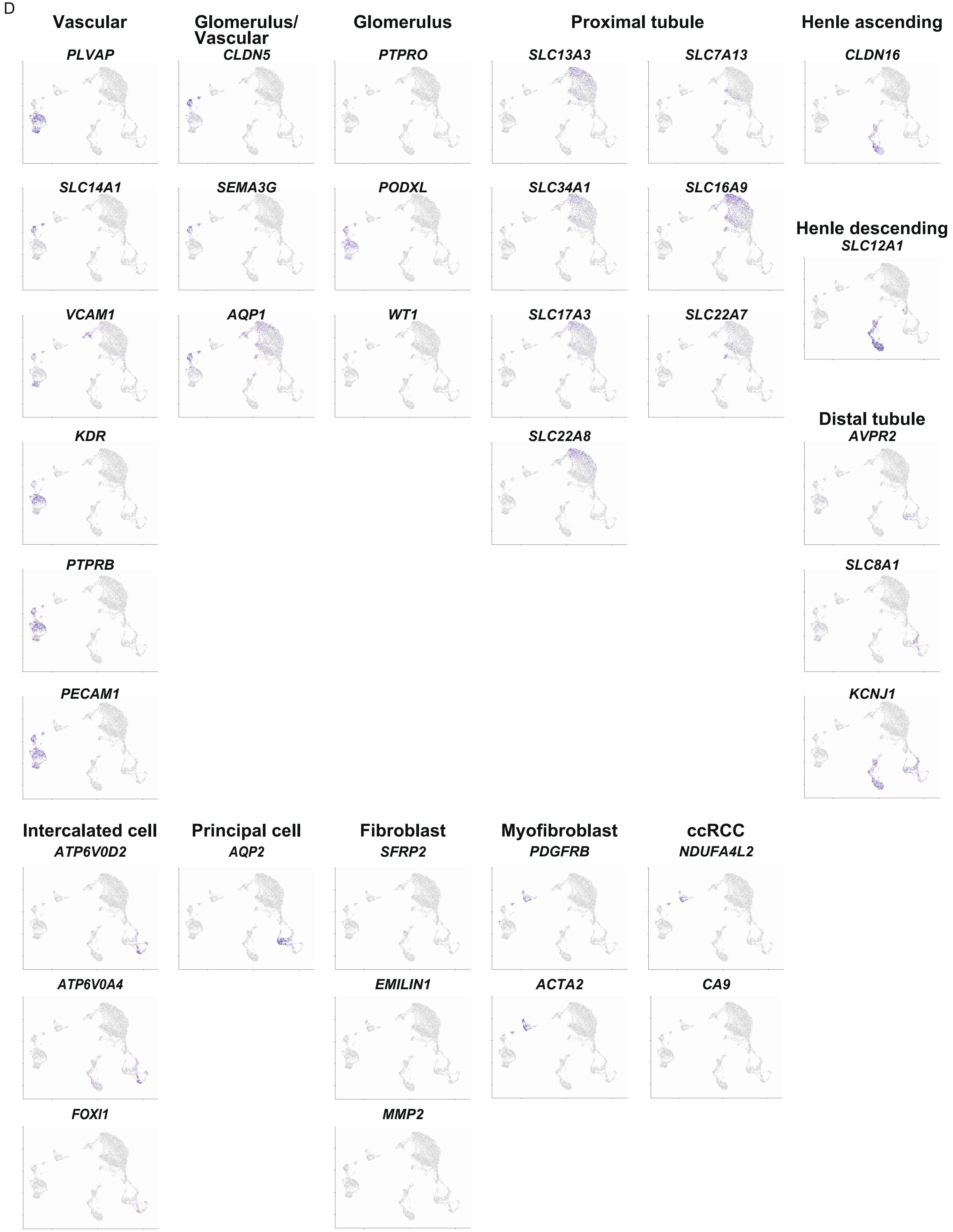
D
Vascular
PLVAP
SLC14A1
VCAM1
KDR
PTPRB
PECAM1
Glomerulus/ Vascular
CLDN5
SEMA3G
AQP1
Glomerulus
PTPRO
PODXL
WT1
Proximal tubule
SLC13A3
SLC7A13
SLC34A1
SLC16A9
SLC17A3
SLC22A7
SLC22A8
Henle ascending
CLDN16
Henle descending
SLC12A1
Distal tubule
AVPR2
SLC8A1
KCNJ1
Intercalated cell
ATP6V0D2
ATP6V0A4
FOXI1
Principal cell
AQP2
Fibroblast
SFRP2
EMILIN1
MMP2
Myofibroblast
PDGFRB
ACTA2
ccRCC
NDUFA4L2
CA9

Figure S3. Creation of normal cell atlas of non-immune cells. Related to Figure 4. (A) UMAP plot shows *CD45* negative non-immune cells from two normal kidney specimens, colored by each specimen. (B) UMAP plot shows *CD45* negative non-immune cells from two normal kidney specimens annotated with marker genes. (C) Heatmap shows gene expressions in annotated clusters. (D) UMAP plots show marker gene expressions in non-immune cells of normal kidney specimens, which were used for creation of normal cell atlas. Abbreviations: ccRCC, clear cell renal cell carcinoma; Henle ascending, ascending limb of the loop of Henle; Henle descending, descending limb of the loop of Henle.

Figure S4

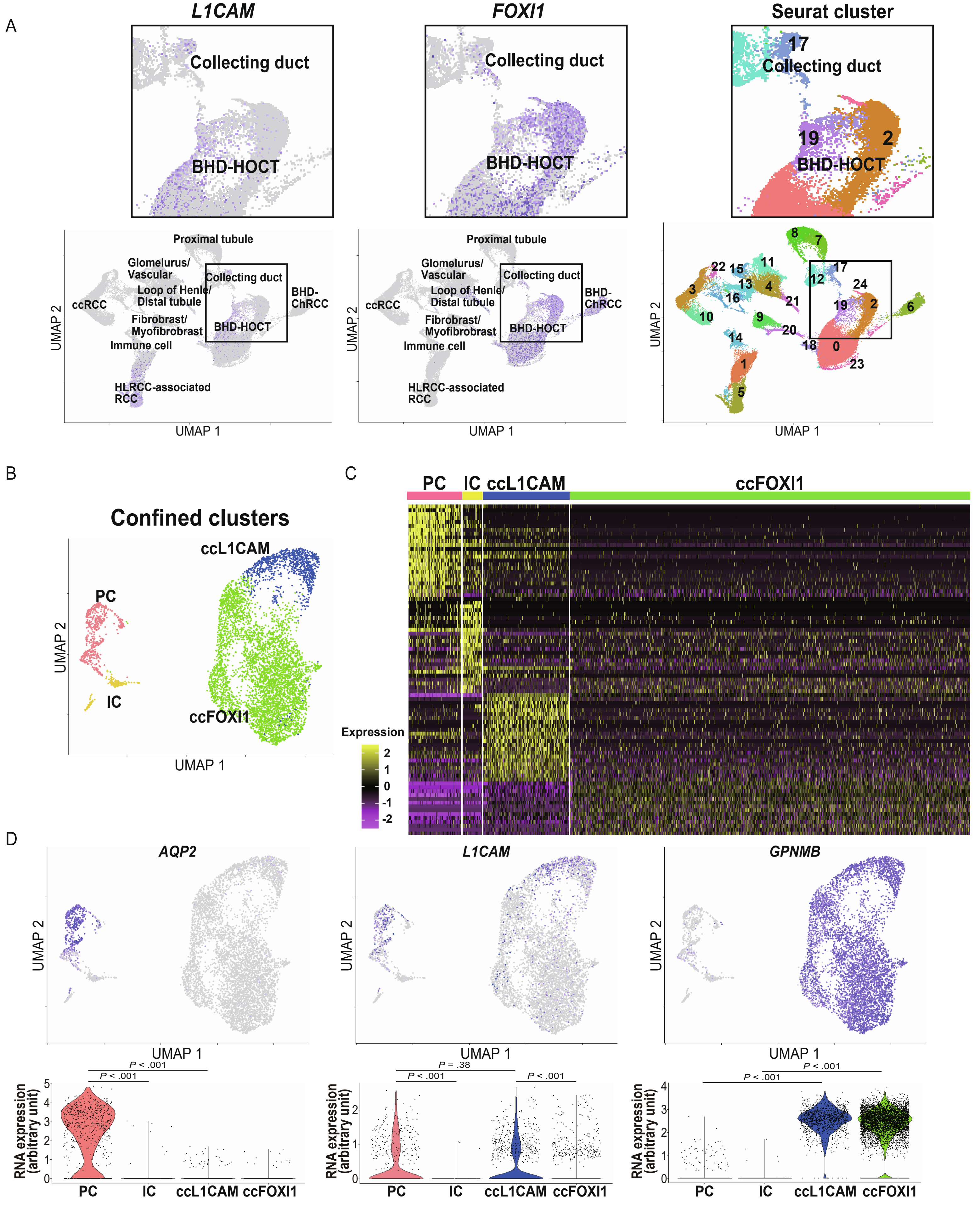

A
L1CAM
FOXI1
Seurat cluster
Collecting duct
BHD-HOCT
17
19
2
Proximal tubule
Glomelurus/ Vascular
Loop of Henle/ Distal tubule
ccRCC
Fibrobrast/ Myofibrobrast
Immune cell
HLRCC-associated RCC
BHD-ChRCC
UMAP 1
UMAP 2
B
Confined clusters
ccL1CAM
PC
IC
ccFOXI1
C
PC
IC
ccL1CAM
ccFOXI1
Expression
2
1
0
-1
-2
D
AQP2
L1CAM
GPNMB
RNA expression (arbitrary unit)
P < .001
P = .38
PC
IC
ccL1CAM
ccFOXI1

Figure S4

E

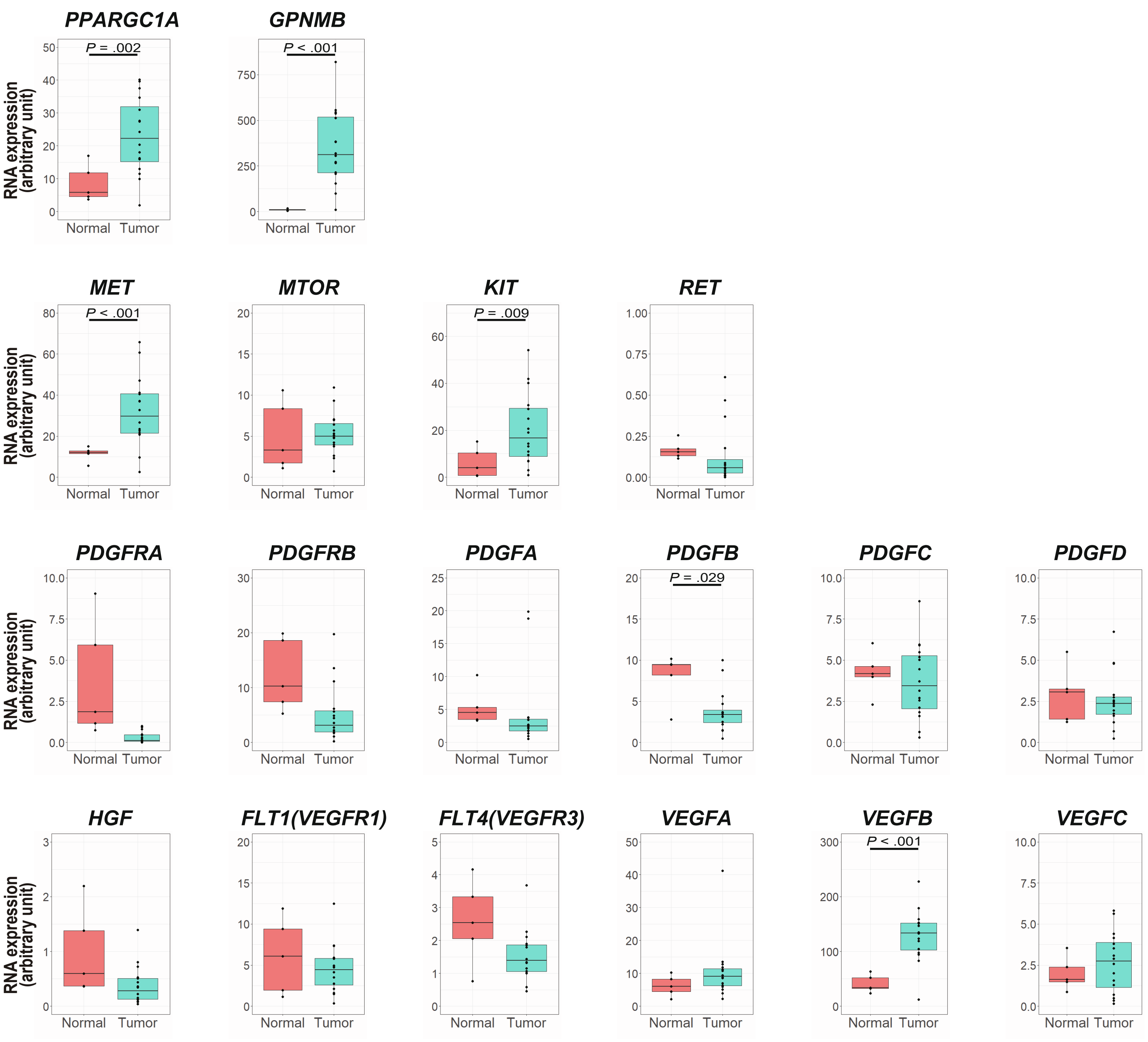
PPARGC1A
GPNMB
MET
MTOR
KIT
RET
PDGFRA
PDGFRB
PDGFA
PDGFB
PDGFC
PDGFD
HGF
FLT1(VEGFR1)
FLT4(VEGFR3)
VEGFA
VEGFB
VEGFC
P = .002
P < .001
P < .001
P = .009
P = .029
P < .001
RNA expression (arbitrary unit)
Normal
Tumor

F

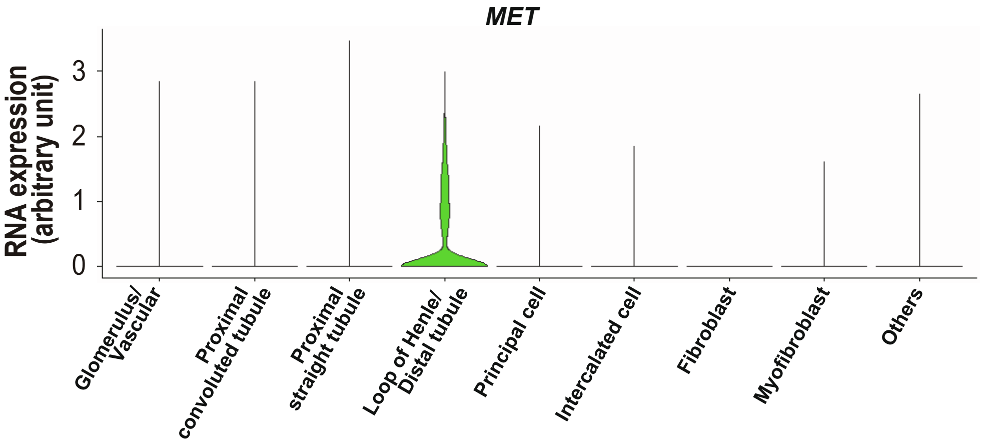
MET
RNA expression (arbitrary unit)
Glomerulus/ Vascular
Proximal convoluted tubule
Proximal straight tubule
Loop of Henle/ Distal tubule
Principal cell
Intercalated cell
Fibroblast
Myofibroblast
Others

Figure S4. Confined clusters used for trajectory analysis of BHD-associated HOCT and normal collecting duct cells and gene expressions in BHD-associated kidney cancer. Related to Figure 4 and Figure 5. (A) UMAP plots show non-immune cells from twelve specimens. Square box is a magnification showing cluster 2, 17 and 19, which were used for trajectory analysis. (B) UMAP plot show principal cells (PC), intercalated cells (IC), a portion of *L1CAM* expressing HOCT cells (confined cluster of L1CAM, ccL1CAM) and a portion of *FOXI1* expressing cells (confined cluster of FOXI1, ccFOXI1). (C) Heatmap of expressions in clusters of Supplementary Figure 4B. (D) Expressions of *AQP2* and *L1CAM*, principal cell marker genes and *GPNMB*, a marker gene for BHD-associated kidney cancer in clusters of Supplementary Figure 4B. (E) RNA-sequencing data of bulk tissues of BHD-associated kidney cancers (n=16) and normal kidneys (n=5) demonstrate increased expressions of *PPARGC1A*, *GPNMB*, *MET*, *KIT* and *VEGFB* in BHD-associated kidney cancers. *P* values are from 2-sided Welch's two sample t-tests. (F) Violin plot shows *MET* expressions in each cluster represented in the normal cell atlas. Abbreviations: BHD-ChRCC, BHD-associated chromophobe renal cell carcinoma; BHD-HOCT, BHD-associated hybrid oncocytic chromophobe tumor; ccFOXI1, confined cluster of *FOXI1* expressing cells; ccL1CAM, confined cluster of *L1CAM* expressing cells; ccRCC, clear cell renal cell carcinoma; HLRCC, HLRCC-associated kidney cancer; IC, intercalated cell; PC, principal cell.

Figure S5

A

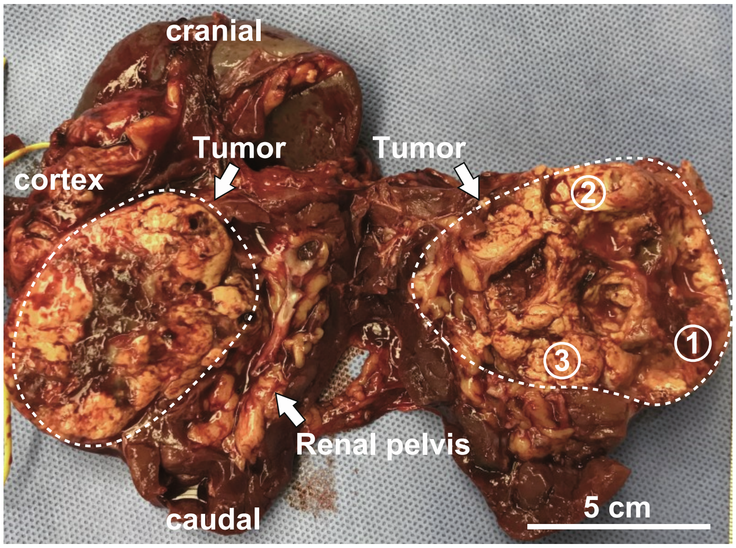

B

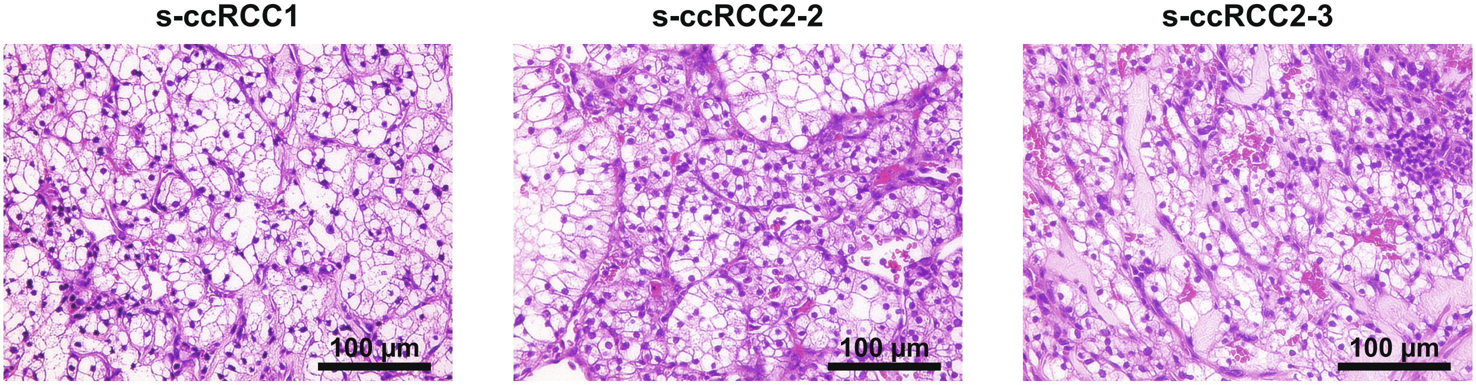

C

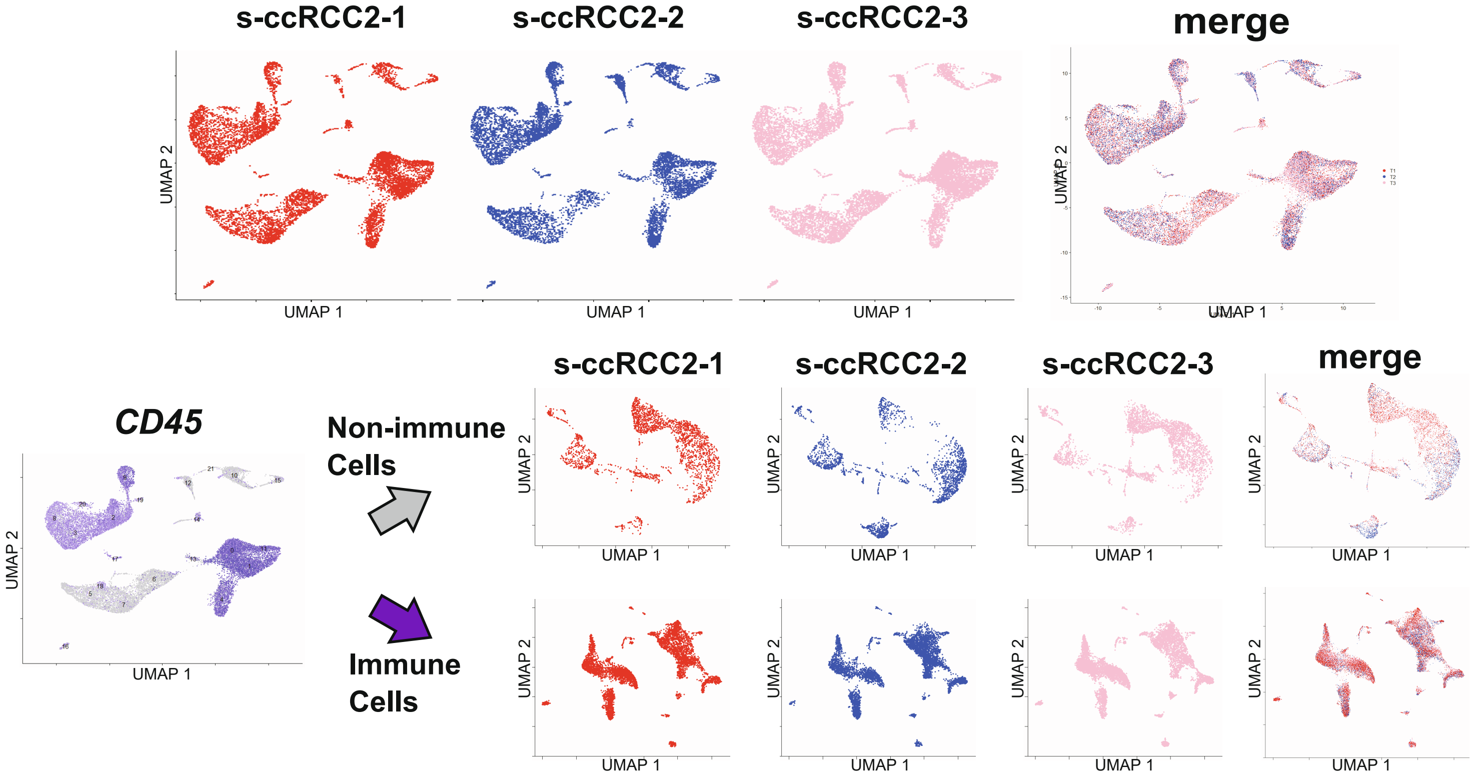

Figure S5

D

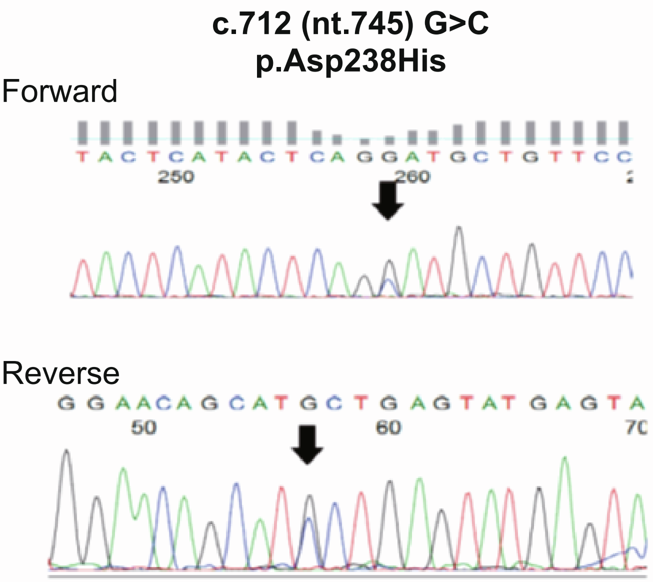

E

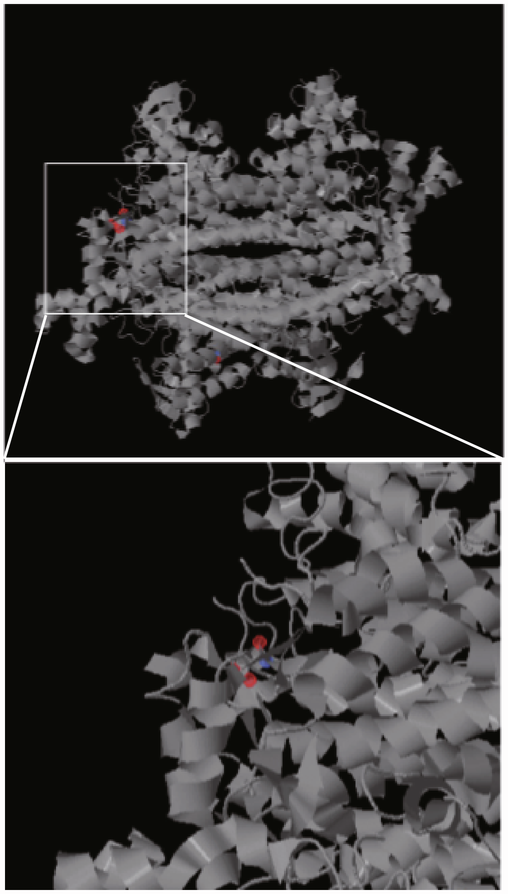

F

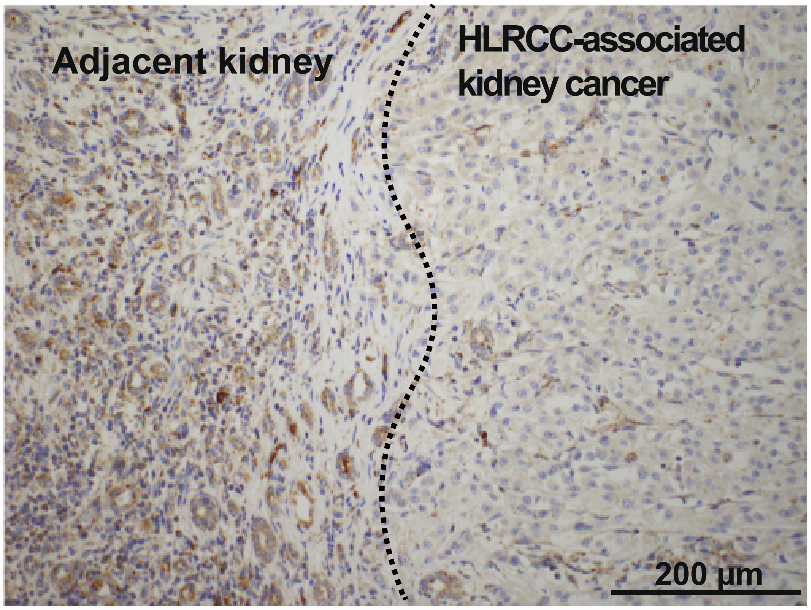

G

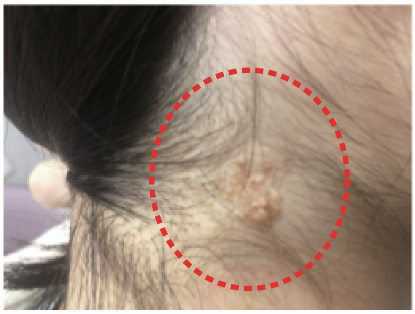

H

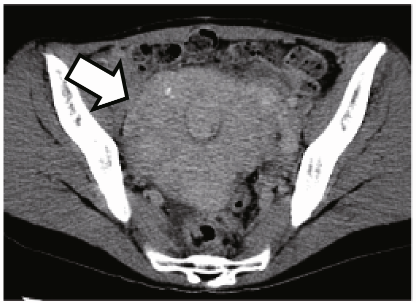

Figure S5. Analysis of multi-site single cell-transcriptome in sporadic clear cell renal cell carcinoma (s-ccRCC2) and Characterization of HLRCC patient. (A) s-ccRCC2-1, s-ccRCC2-2 and s-ccRCC2-3 were biopsied from lesion 1,2 and 3 of s-ccRCC2, respectively. (B) All of histologies of s-ccRCC2-1, s-ccRCC2-2 and s-ccRCC2-3 were confirmed to be clear cell RCC. Image shows a 400x magnification of hematoxylin and eosin staining. Scale bars represent 100 μm. (C) No transcriptomic intratumor heterogeneity (tITH) was observed in multi-site biopsied samples of s-ccRCC2. (D) Germline missense variant in Fumarate hydratase (FH) gene was detected in HLRCC patient. (E) 3D image shows the location of Asp238 in FH protein (red highlighted). (F) Immunohistochemistry shows loss of FH protein in HLRCC-associated kidney cancer. Scale bars represent 200 μm. (G) Picture shows skin lesions (red dotted circle) in the neck of HLRCC patient. (H) Computed tomography shows uterine fibroid (arrow) in HLRCC patient. Abbreviations: s-ccRCC, sporadic clear cell renal cell carcinoma.
